# Cre recombinase microinjection for single-cell tracing and localised gene targeting

**DOI:** 10.1242/dev.201206

**Published:** 2023-02-03

**Authors:** Miquel Sendra, Juan de Dios Hourcade, Susana Temiño, Antonio J. Sarabia, Oscar H. Ocaña, Jorge N. Domínguez, Miguel Torres

**Affiliations:** ^1^ Cardiovascular Regeneration Program, Centro Nacional de Investigaciones Cardiovasculares, CNIC, 28029 Madrid, Spain; ^2^ Transgenesis Unit, Centro Nacional de Investigaciones Cardiovasculares, CNIC, 28029 Madrid, Spain; ^3^ Department of Experimental Biology, Faculty of Experimental Sciences, University of Jaén, 23071 Jaén, Spain; ^4^ Fundación MEDINA, Centro de Excelencia en Investigación de Medicamentos Innovadores en Andalucía, Avenida del Conocimiento 34, 18016 Granada, Spain

**Keywords:** Fate mapping, Clonal analysis, Labelling, Cre recombinase microinjection, Mouse

## Abstract

Tracing and manipulating cells in embryos are essential to understand development. Lipophilic dye microinjections, viral transfection and iontophoresis have been key to map the origin of the progenitor cells that form the different organs in the post-implantation mouse embryo. These techniques require advanced manipulation skills and only iontophoresis, a demanding approach of limited efficiency, has been used for single-cell labelling. Here, we perform lineage tracing and local gene ablation using cell-permeant Cre recombinase (TAT-Cre) microinjection. First, we map the fate of undifferentiated progenitors to the different heart chambers. Then, we achieve single-cell recombination by titrating the dose of TAT-Cre, which allows clonal analysis of nascent mesoderm progenitors. Finally, injecting TAT-Cre to *Mycn^flox/flox^* embryos in the primitive heart tube revealed that *Mycn* plays a cell-autonomous role in maintaining cardiomyocyte proliferation. This tool will help researchers identify the cell progenitors and gene networks involved in organ development, helping to understand the origin of congenital defects.

## Teaser

**Highlighted Article:** TAT-Cre microinjection can be used to trace cell lineages and edit genes at custom anatomical locations in postimplantation mouse embryos, allowing prospective clonal analysis and genetic mosaic studies in progenitor cell populations.

## INTRODUCTION

During development, stem cells diversify and organise to build complex systems. Knowing the origin of the cell progenitors involved in each specific process helps us to understand how organs are built. Since the end of the 19th century, biologists have traced cell lineages to identify within heterogeneous progenitor populations those that contribute to specific body structures and functions (Whitman, 1898). For this, groups of cells are labelled and tracked to determine which tissues they contribute to (Lawson et al., 1987; Tam and Zhou, 1996). Furthermore, analysing the progeny of single cells, also known as clonal analysis, allows inferring when they commit to different fates (Padrón-Barthe et al., 2014; Tu and Johnson, 2011).

A variety of cell tracing techniques are now available (VanHorn and Morris, 2021). In prospective methods such as dye injection, grafting or retroviral infection, researchers choose the stage and location of the targeted cells, the progeny of which is tracked over time. They always require direct manipulation of the sample. For viviparous embryos, this implies that its applicability is limited to the stages that can be grown *ex utero*. In retrospective methods, the labelling is achieved through genetic drivers, and the resulting progenies are only examined at the endpoint. Unlike prospective methods, they do not require physical manipulation or sample culture. This makes them highly scalable, recording thousands of cell lineages at the same time if coupled with single-cell transcriptomics (Spanjaard et al., 2018; Chan et al., 2019). Although genetic inducible systems can restrict the labelling to specific stages and cell domains, the time and location of induction are only approximate or unknown (Petit et al., 2005). For this reason, prospective tracing approaches are preferred in experiments that require precise temporal and spatial information about the cells being labelled.

Prospective tracing allows mapping the contribution of undifferentiated embryonic regions to specific organs and tissues. By labelling groups of cells using lipophilic dye microinjection, the germ layers and organ primordia were mapped to different regions of the epiblast in bilaterian embryos (Kinder et al., 1999; Lawson et al., 1987; Dale and Wardle, 1999; Kimmel et al., 1990; Hatada and Stern, 1994). Labelling single cells is more challenging. Individual cells can be labelled by intracellular injections of compounds that cannot diffuse from one cell to another, such as horseradish peroxidase, fluorescent dextran conjugates or nucleic acids that encode a reporter gene (Lee et al., 1994; Serbedzija et al., 1989). However, beyond blastula stages, the embryo cells become progressively smaller, making the experiment more complex and time-consuming. This is detrimental to mammalian embryos, which are sensitive to manipulation and culture *ex utero* (Nagy et al., 2014).

In post-implantation mouse embryos, intracellular injection of horseradish peroxidase by iontophoresis has been used to prospectively trace single cells in the epiblast (Lawson et al., 1991). However, most injections labelled more than one cell. To develop a reliable alternative for prospective clonal analysis in the mouse model, we took advantage of the Cre-loxP system. By microinjecting cell-permeant Cre recombinase in embryos carrying floxed reporter alleles, we targeted custom embryonic stages and anatomical locations. In this way, we were able to fate map splanchnic mesoderm progenitors that contribute to the looping heart. Next, titration of the TAT-Cre dose produced single-cell labelling, allowing clonal analysis in the nascent mesoderm. Finally, the injection of TAT-Cre into conditional knockout (KO) embryos for *Mycn* revealed its cell-autonomous role in embryonic cardiomyocyte proliferation. The method we present here uses equipment and genetic systems that are widely available in molecular biology laboratories, offering a strategy for fate mapping, clonal analysis and localised genetic manipulations.

## RESULTS AND DISCUSSION

### TAT-Cre recombinase microinjection allows lineage tracing in mouse embryos

**Fig. 1.**
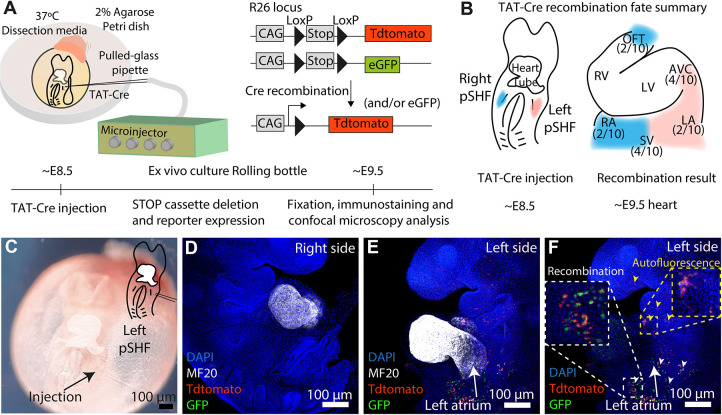
**TAT-Cre microinjection in E8.5 embryos recapitulates the fate map of posterior second heart field (pSHF) progenitors.** (A) Experiment setup. (B) Posterior second heart field (pSHF) contribution (*n*=10 embryos from two litters). (C-F) Result of a left pSHF injection. MF20 (D,E) marks the heart tube. White and yellow arrowheads point to recombination and unspecific signal, respectively (F). AVC, atrioventricular canal; LA, left atrium; LV, left ventricle; OFT; outflow tract; RA, right atrium; RV, right ventricle; SV, sinus venosus.

We developed a strategy to trace the progeny of groups of cells at custom anatomical locations in mouse embryos. We used pneumatic microinjection of membrane-permeable Cre recombinase protein (TAT-Cre) in embryos carrying LoxP-flanked STOP cassettes preceding fluorescent reporter genes in the *Rosa26* locus (Madisen et al., 2010; Sousa et al., 2009). We then cultured the embryos *ex vivo* and detected the progeny of the recombined cells (Fig. 1A).

To test TAT-Cre microinjection for lineage tracing, we targeted the posterior second heart field (pSHF) (Galli et al., 2008) in ten approximately embryonic day (E) 8.5 embryos and analysed the contribution of Cre-recombined cells after 24 h of *ex vivo* culture. Consistent with previous lipophilic dye microinjection studies (Domínguez et al., 2012; Galli et al., 2008), the E8.5 pSHF contributed to the atria (A), the atrioventricular canal (AVC), the sinus venosus (SV) and the outflow tract (OFT) of the E9.5 heart (Fig. 1B). Notably, microinjection of the left pSHF in E8.5 embryos produced Tomato+ and GFP+ cells only in the left SV and AVC at E9.5, without recombination in the right SV or OFT (Fig. 1C-F), recapitulating previous data showing that the left pSHF contributes ipsilaterally to the heart tube (Domínguez et al., 2012). These data show that TAT-Cre recombinase can be used as a lineage tracing tool in mouse embryos.

### Titration of TAT-Cre recombinase for clonal analysis

Next, we considered the use of TAT-Cre microinjection for clonal analysis. To that end, we adjusted TAT-Cre dose to produce clones – groups of labelled cells derived from a single progenitor cell. A good dose is a compromise between enough recombination efficiency and targeting only one cell at a time: A high dose is more likely to achieve efficient recombination but also has more potential for labelling more than one progenitor. On the contrary, a low dose might produce recombination too rarely. We tested three TAT-Cre doses: dose 1 consisting of ∼0.065 pl of undiluted TAT-Cre solution, dose 1/2, diluted to half, or dose 1/4, diluted to the fourth with buffer (Materials and Methods).

**Fig. 2.**
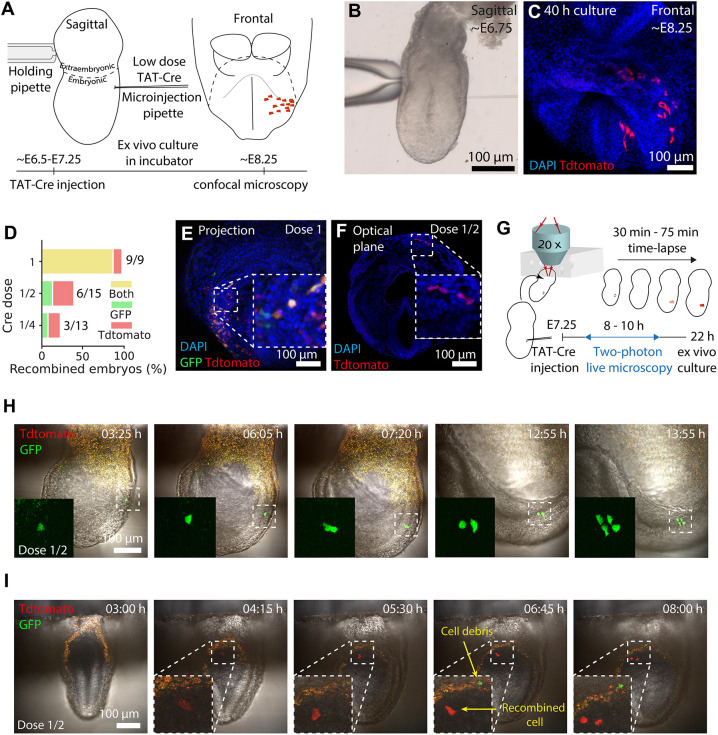
**TAT-Cre titration for clonal analysis.** (A) Microinjection setup. (B,C) An early-streak embryo at injection (B) and after culture (C). (D) Proportion of fluorescent embryos on each TAT-Cre dose tested. (*n*=37 embryos, six litters). (E,F) Microinjected embryos after culture. (G) Live imaging setup. (H,I) Time series (300 μm, H; 20 μm, I) projection for microinjected embryos. In I, cell debris appears at 4:15 but it is outside the 20 μm projection. Time is reported as h:min.

First, we tested the efficiency of each dose. We microinjected TAT-Cre in the posterior region of ∼E6.5-E7.25 embryos (in the nascent mesoderm or the ectoderm/epiblast in case of pre-streak embryos) and cultured them statically (Fig. 2A-C). To reach the stage of early somitogenesis, we cultured ∼E7.0-E7.25 embryos for 24 h and ∼E6.5-E7.0 embryos for 40 h. We used a zygotic microinjection setup (Fig. S2; Movie 1), which allowed us to deliver low and consistent volumes of TAT-Cre solution. The highest dose tested (1) labelled all microinjected embryos, whereas lower doses (1/2 and 1/4) were less efficient in producing recombination (Fig. 2D-F).

Second, we tested whether the lower doses were more likely to produce clones using three complementary approaches: live-imaging cell tracking, the two-reporter strategy statistical method and distribution-inference of cell counts.

We cultured and time-lapse imaged ten TAT-Cre microinjected embryos (Fig. 2G; Movie 2) using either dose 1 or dose 1/2. Back-tracking labelled cells allowed us to distinguish whether they derived from single (clonal) or multiple (polyclonal) recombination events. Although dose 1 generated polyclonal events, dose 1/2 labelled only 2/5 of the embryos (Fig. S3A), each containing a single recombined cell (Fig. 2H,I; Movie 2).

Next, the two-reporter strategy (Lioux et al., 2020; Padrón-Barthe et al., 2014) allowed us to estimate how often a monocolour group of cells can originate from two independent recombinations, as the frequency of bicolour events in a collection of samples is directly proportional to its polyclonality (Materials and Methods). Live-imaged embryos showed that dose 1 is more likely to recombine multiple cells than dose 1/2, and therefore yields more bicolour groups of labelled cells (Fig. S3A). Similarly, the two lower doses (1/2 and 1/4) lacked bicolour groups of labelled cells in fixed embryos analysed at the experimental endpoint (Fig. 2D), suggesting a low probability of polyclonal events.

Then we tested whether the size of each group of labelled cells was consistent with a clonal origin (Zhang et al., 2021). The number of cells recombined in fixed embryos analysed at the experimental endpoint for doses 1/2 and 1/4 was comparable with those obtained in live imaging tracked clones (Fig. S3B) and (Fig. S3C,D), which underwent cell division two to three times (cell numbers range from 2^2^ to 2^3^). This is consistent with the cell division rates in live tracked cells (6:00 and 6:20 h:min in ectoderm; 7:05 and 7:25 h:min in mesoderm, Fig. S3E,F) and previously reported rates in early gastrulating embryos (Solter et al., 1971). Overall, both 1/2 and 1/4 ensured a high probability of clonality. We chose dose 1/2 as it yielded a higher recombination efficiency.

### Spatial and temporal resolution of TAT-Cre recombination

In addition to the efficiency and number of cells labelled, we studied where and when recombination occurs after injection. Unlike other methods using intracellular microinjections of undiffusible compounds, which can only be performed in cohesive cell sheets (Lawson et al., 1991, 1986), TAT-Cre injections can also be applied to mesenchymal tissues. Previous clonal analyses in mouse embryos thus only targeted epithelial layers, such as the epiblast and endoderm, whereas TAT-Cre can also target mesenchymal tissues such as the nascent mesoderm.

However, unlike fluorescent dyes, one cannot visualise TAT-Cre in the targeted cells at the time of injection. This represents a limitation. To determine the range of recombined cells after injection, we mapped the positions of independently recombined live-imaged cells at the first time that fluorescent reporters were detected. The distance between cells ranged from ∼50 to ∼80 μm (Fig. S4A,B). Although these results suggest a wide range of possible recombination around the injection site, cells might also move apart from each other from the time of recombination to the first detection of the reporter. In fact, extensive cell mixing and migration have been reported previously in gastrulating embryos (Ichikawa et al., 2013; Saykali et al., 2019).

To assess how long it takes from injection to labelling, we then quantified the dynamics of fluorescence intensity in live imaging data (Fig. S5B). All GFP and Tdtomato cells tracked were detectable within the first 7 h, although GFP events appeared earlier (Fig. S5C). This may have been due to differences in the time that TAT-Cre takes to recombine one or the other reporter cassette or to differences in laser/detector efficiency during image acquisition. In support of the latter, TAT-Cre has been shown to produce recombination within the first 1 h when administered to cell cultures (de Vasconcelos et al., 2020). The delay between injection and reporter detection makes this method less time-defined than others that are based on injected fluorescent compounds. However, fluorescent compounds dilute on each cell division, limiting their application to study lineages over three to four cell divisions (Petit et al., 2005; Mobbs et al., 1994; Buckingham and Meilhac, 2011). Here, using the *Rosa26* genetic reporter, after recombination of TAT-Cre the reporters are permanently expressed, making it possible to study highly proliferative cells without losing signal intensity.

The delay between injection and detection is compatible with the spread of recombined cells from a narrower initial distribution. To assess the range of cells actually exposed to TAT-Cre, we immunostained embryos against the 6xHis-tag of TAT-Cre immediately after dose 1 microinjection (Fig. S4C). Measurement of the distance between TAT-Cre-positive cells indicated local incorporation in the proximity of the injection site, ranging from 0 to ∼30 μm (Fig. S4B). The nuclear localisation of TAT-Cre indicates its availability for recombination immediately after injection (Fig. S4C).

Associated with the injection site, we often observed cell debris, which suggests that the needle kills injected cells, while surviving surrounding cells may uptake TAT-Cre and recombine (Fig. S6A,B). As a note of caution, injection of TAT-Cre for local labelling should be done in embryonic layers and not in cavities, which can lead to spread recombination (Fig. S6C,D).

In summary, the results show that recombinations occur within a narrow range from the injection site and that the reporter signal can be detected within the first 7 h.

### Prospective clonal analysis of nascent mesoderm progenitors

As a proof of concept for clonal analysis, we microinjected dose 1/2 of TAT-Cre to 41 additional embryos from pre-streak (∼E6.5) to late bud (∼E7.25) stages (Downs and Davies, 1993) in the nascent mesoderm (or the posterior epiblast in the case of pre-streak embryos) and cultured them. After discarding eight undeveloped embryos, we detected 19 embryos, each containing a fluorescent group of cells (Fig. S7A-D). Of these, one contained both GFP and Tdtomato cells and the rest were monocolour (Fig. S7E). The use of the two-reporter strategy (Lioux et al., 2020; Padrón-Barthe et al., 2014) allowed us to estimate that monocolour groups of labelled cells in our embryo collection had a 93% chance of being clonal (Fig. S7F; Materials and Methods), which doubles the clonal rate obtained with horseradish peroxidase microinjection (Lawson et al., 1991). Likewise, the number of cells per embryo was consistent with their clonal origin (Fig. S7G).

**Fig. 3.**
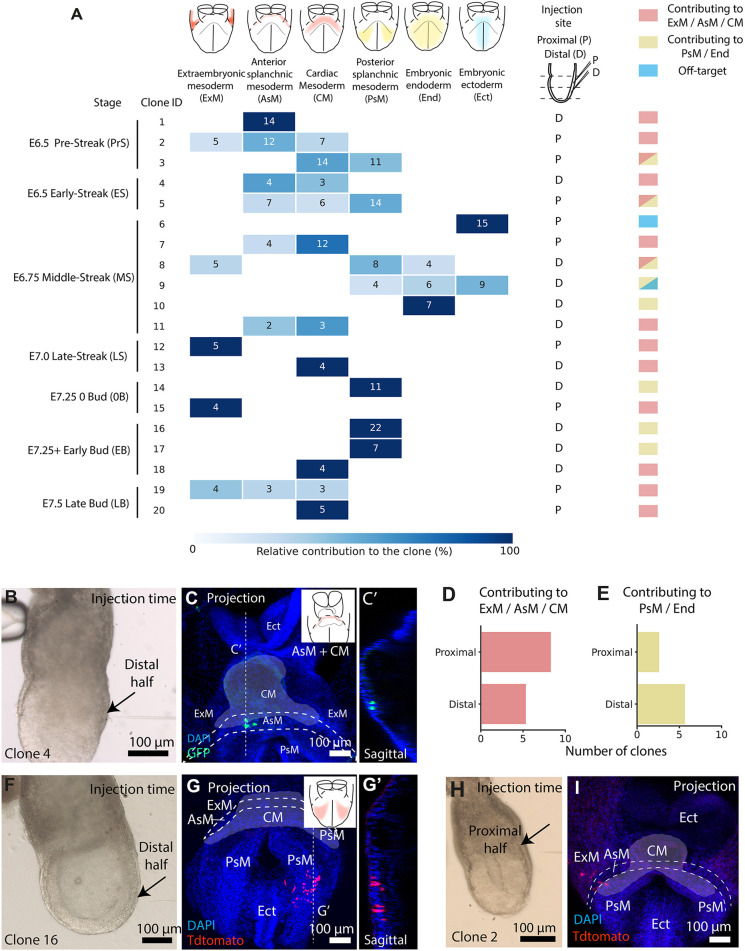
**TAT-Cre microinjection allows prospective clonal analysis of nascent mesoderm.** (A) Contribution of dose 1/2 TAT-Cre-induced clones. Squares contain cell counts (*x*-axis, clone; *y*-axis, location. *n*=19 embryos, eight litters). (B-C′) Injected embryo (B) and its resulting clone (C). C′ shows a sagittal plane of C. (D,E) Distal and proximal injections contributing to ExM, AsM and CM (*n*=14 embryos) (D) and to the PsM and End (*n*=8 embryos) (E). (F-G′) Injected embryo(F) and its resulting clone (G). G′ shows a sagittal plane of G. (H,I) Injected embryo (H) and its contribution (I) to embryonic and extra-embryonic compartments (*n*=2 embryos). Cardiac mesoderm is highlighted with white shading and embryonic compartments annotated following abbreviations in A.

During gastrulation, cells from the epiblast ingress the primitive streak and form the nascent mesoderm, becoming progressively restricted to different fates (Tam et al., 1997). Cell labelling and grafting experiments showed that the first cells ingress at the proximal end of the primitive streak and give rise to extra-embryonic compartments, followed by cardiac and cranial progenitors (Tam et al., 1997; Lawson et al., 1987, 1991; Lawson and Pedersen, 1992). As the primitive streak extends distally, definitive endoderm and other posterior mesoderm progenitors are generated (Tzouanacou et al., 2009). Consistent with this, TAT-Cre injections in the proximal half of the nascent mesoderm contributed more to the extra-embryonic and cardiac mesoderm regions, whereas distal injections contributed more to the posterior mesoderm domains and endoderm (Fig. 3A-G). Interestingly, two of the clones contributed both to embryonic and extra-embryonic compartments (Fig. 3H,I), suggesting that the recently discovered multipotent progenitors (Tyser et al., 2021; Zhang et al., 2021) may be present in the early nascent mesoderm.

These results also showed the limitations of TAT-Cre for clonal analysis. We observed that aiming the injection at the mesoderm can also cause off-target recombination in neighbouring ectodermal cells. For example, clone 5 contributed to the ectoderm, which is an unlikely fate for a mesodermal cell (Fig. 3A). Live imaging also shows that an injection aimed at the mesoderm can cause recombination in the ectoderm (Fig. S3A, embryo e007). Furthermore, the mixed contribution to both target and off-target layers confirms the possibility of polyclonal recombination (clone 9, Fig. 3A).

TAT-Cre microinjection relies on low recombination frequencies to yield high chances of single-cell targeting in specific layers. The low occurrence of off-target and polyclonal recombination could be adjusted further according to the needs of the specific scientific question by titrating the concentration of the injected TAT-Cre. In summary, these results show that TAT-Cre clonal analysis can be performed using instrumentation that is commonly available in molecular biology laboratories and reproduce previous experiments.

### Mosaic Cre/loxP-mediated genetic knockout at custom embryonic stages and anatomical locations

Cre-lox genetic modifications have transformed biomedical research. Modification of *LoxP* engineered loci is now available for a variety of mouse lines that express Cre- or tamoxifen-inducible CreERT2 under specific promoters. However, this application is limited to embryonic domains with available Cre drivers. By microinjecting TAT-Cre into floxed mouse models, this tool can be extended to nearly any embryonic location and stage of early development.

To validate this application, we took advantage of the iSuRe-Cre system, a tool to reliably induce and report Cre-mediated genetic modifications (Fernández-Chacón et al., 2019). In brief, iSure-Cre is an inducible dual reporter-Cre mouse allele, which activates permanent Cre expression in reporter-expressing cells, ensuring that they completely recombine any floxed alleles.

**Fig. 4.**
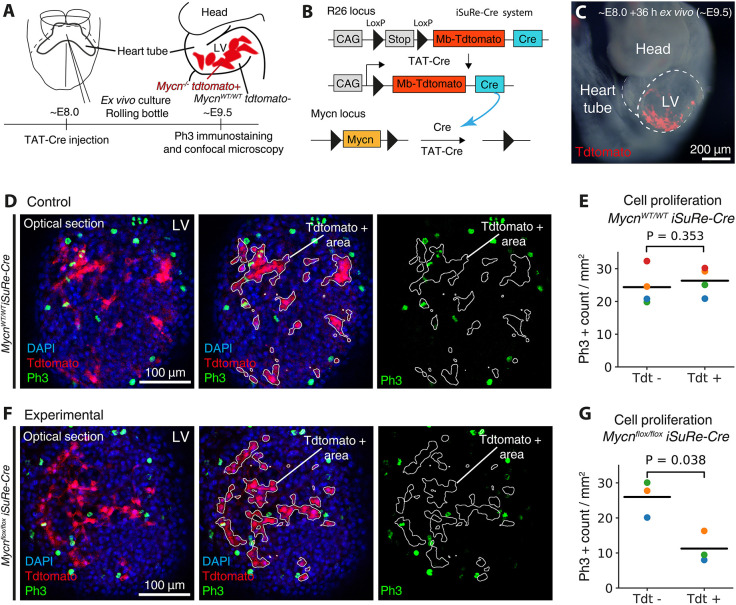
**Mosaic TAT-Cre *Mycn* knockout in embryonic cardiomyocytes.** (A) Setup to induce *Mycn^−/−^* Tdtomato+ cells and quantify their proliferation. (B) Deleting *Mycn* using the iSure-Cre and *Mycn*-floxed alleles. (C) Embryo after TAT-Cre injection and culture. (D,F) Control (D) and experimental (F) E9.5 embryos immunostained with Ph3. (E,G) Density of Ph3+ cells in Tdtomato− (Tdt−, unrecombined) and Tdtomato+ (Tdt+, recombined) domains in wild-type (WT, E) and *Mycn*-floxed (G) embryos, normalised by area (horizontal lines, mean; coloured points, individual embryos; *n*=4 WT and *n*=3 *Mycn*-floxed embryos, two litters; unpaired two-tailed *t*-test of two related samples). LV, left ventricle.

*Mycn* is essential to maintain cell proliferation in *Nkx2-5*+ embryonic cardiomyocytes (Muñoz-Martín et al., 2019), and we used this model to determine whether injected TAT-Cre could recapitulate a reduction in cell proliferation in genetic mosaics by locally deleting *Mycn*. For this, we microinjected TAT-Cre into the primitive heart tube of control (*Mycn^wt/wt^; iSuRe-Cre^+/−^*) and *Mycn*-floxed (*Mycniptflox/flox; iSuRe-Cre+/−*) embryos (Fig. 4A-C). After 36 h of *ex vivo* culture, we immunostained for the proliferation marker phospho-histone H3 (Ph3). Counting the density of Ph3+ cells in TAT-Cre targeted (tdt+) and untargeted (tdt−) domains revealed that, although proliferation was unaltered in the recombined cells of control embryos (Fig. 4D,E), it was reduced in the *Mycn*-KO cells of the experimental embryos (Fig. 4F,G). These results show that the *Mycn* role in cardiomyocyte proliferation is cell-autonomous and that LoxP modifications can be performed in custom embryonic locations by directly microinjecting TAT-Cre.

Currently, available approaches include the microinjection of viruses; however, this yields variable outcomes that are influenced by tropism and off-target insertions (Artegiani and Calegari, 2013; Mangold et al., 2021). Alternatively, targeted electroporation of DNA constructs can produce consistent and spatially restricted genetic changes (Pierreux et al., 2005; Mazari et al., 2014). However, these modifications are transient and can only be targeted to exposed cell layers – for example, in early post-implantation embryos, electroporation is only efficient in endodermal cells. In this sense, TAT-Cre injection is more versatile than previous prospective techniques used in mouse embryos.

On the other side, TAT-Cre injection has limitations. As the distance between *LoxP* sites (Koike et al., 2002) and their chromosomal location (Vooijs et al., 2001) can affect sensitivity to recombination, different floxed alleles may require adjusting the TAT-Cre doses reported here. Although low volumes of TAT-Cre were sufficient to induce recombination in our experiments, they may not suffice in extremely insensitive *LoxP* alleles. This is especially crucial when aiming to target a high percentage of cells.

In summary, TAT-Cre microinjection is a versatile tool for fate mapping, clonal analysis, and local gene modifications in mouse embryos. This protocol relies on equipment that is available in most developmental biology laboratories, making it accessible. Provided proper dissection and *ex vivo* culture setups, this tool will allow to study cell lineages and dissect gene roles in developing organs.

## MATERIALS AND METHODS

### Mouse strains

*ROSA26R^CAG-Tdtomato^* (Madisen et al., 2010), *ROSA26R^CAG-GFP^* (Sousa et al., 2009), *Polr2a^CreERT2^*(RERT) (Guerra et al., 2003), *iSuRe-Cre* (Fernández-Chacón et al., 2019) and *Mycn* floxed (Knoepfler et al., 2002) have been previously described. For clonal analysis, *ROSA26R^CAG-Tdtomato^* homozygous mice were crossed with *ROSA26R^CAG-GFP^* homozygous mice. For localised *Mycn* deletion, heterozygous *iSuRe-Cre* mice were mated with *Mycn* homozygous mice. All animal procedures were conducted in accordance with CNIC Ethics Committee, Spanish laws and the EU Directive 2010/63/EU for the use of animals in research. All mouse experiments were approved by the CNIC and Universidad Autónoma de Madrid Committees for ‘Ética y Bienestar Animal' and the area of ‘Protección Animal' of the Community of Madrid with reference PROEX 144.1/21.

### Embryo isolation and culture

Mouse embryos were harvested and dissected in pre-equilibrated dissection medium: DMEM supplemented with 10% foetal bovine serum, 25 mM HEPES-NaOH (pH 7.2), penicillin (50 μg/ml) and streptomycin (50 μg/ml). Embryos from E6.5 to E7.5 were cultured in static conditions in a hypoxic chamber incubator (Fig. S2) in culture media: 50% Janvier Labs Rat Serum Sprague Dawley RjHan SD male only and 50% DMEM FluoroBrite (Thermo Fisher Scientific, A1896701), penicillin (50 μg/ml) and streptomycin (50 μg/ml). Embryos from E8 onward were cultured in an embryo roller culture system (Fig. S1A,B), with 75% rat serum and 25% T6 solution (Whittingham, 1971). Temperature was set to 37°C, with 5% O_2_ and 7% CO_2_ concentration for E6.5-E8 embryos (González et al., 2021), whereas embryos from E8 onward were cultured in 20% O_2_.

### TAT-Cre recombinase microinjection for fate mapping and gene ablation

For fate mapping or local gene ablation experiments, E7.5 to E8.5 embryos were microinjected with undiluted TAT-Cre recombinase (SCR508, Sigma-Aldrich) using a pneumatic microinjector (Narishige IM 300 Microinjector) (Fig. S1C,D). Needles used for these microinjections were made using a Sutter Instrument Co. puller (Fig. S2D). The conditions for microinjections were 9 psi of injection pressure during 40 ms. To estimate the volume delivered per injection, we used the same settings to deposit drops of the injected solution in paraffin oil and measured its diameter as previously described (Rosen et al., 2009). With a resulting diameter of 90 μm, and using the equation of a sphere 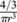, we estimated the volume of the drop to be 0.38 nl. As the TAT-Cre solution contained 1000 enzymatic units (U) per ml, each pulse delivered 3.8·10^−4^ U of TAT-Cre. Embryos were held frontally in an agarose-coated Petri dish. A single pulse per embryo was delivered for pSHF fate mapping assays. Two pulses were delivered for *Mycn* floxed assays.

### TAT-Cre recombinase microinjection for clonal analysis

For single-cell lineage tracing, embryos from E6.5 to E7.5 were microinjected with TAT-Cre recombinase (SCR508, Sigma-Aldrich) in IDTE (pH 7.5) 1× buffer (10 mM Tris, 0.1 mM EDTA) using a Zeiss Observer D1 microscope; Transferman Nk2 micromanipulators and FemtoJet injection pump (Eppendorf) (Fig. S2A,B). Microinjection needles were made in a Sutter Instrument Co. puller and forged to get a 2 μm gauge needle (Fig. S2C-E).

Embryos were placed into a Petri dish with dissection medium coated with paraffin oil (Nidoil, Nidacon, VNI0049) to avoid evaporation. Later, the embryos were positioned laterally so that the anterior and posterior sides of the embryo were on the left and right sides of the user view, respectively.

Embryos were then held by the extra-embryonic region using a pulled glass-rounded pipette. Then the tip of a microinjection needle loaded with TAT-Cre solution was inserted at the posterior side of the embryo until the endodermal layer was crossed. Then 800 hPa output pressure and 100 hPa compensation pressure were applied for 2 s before removing the needle. The injected volume was estimated using Poiseuille Law. Assuming that the TAT-Cre solution behaved as a laminar fluid and approximating the injection needle to a cylinder geometry, we applied the following equation to calculate the flow rate, as volume of TAT-Cre solution per second:

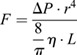
where: *F* is flow rate, in *m*^3^/*s*_;_ Δ*P* is pressure difference in *Pa* (*Pi*=800 hPa and *Pc*=100 hPa for *Pi*, injection pressure and *Pc*, compensation pressure); *r* is radius in *m* (injection needle radius, 2.5 μm); *η* is viscosity of the microinjected solution in *Pa*/*s* (0.001 *Pa*/*s* for 1:1 IDTE buffer: TAT-Cre at 25°C); *L* is length in *m* (injection needle length, 75 mm).

By using an injection time of 2 s, we estimated a microinjection volume of 0.104 pl per embryo. To empirically calculate this volume, we used the same injection settings to deposit drops of the injected solution in paraffin oil and measured its diameter as previously described (Rosen et al., 2009). With a resulting diameter of 5 μm, and using the equation of a sphere 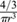, we estimated the volume of the drop to be 0.065 pl. Provided the TAT-Cre solution contained 1000 enzymatic units (U) per ml, and assuming an injection volume of 0.065 pl, the estimated injected doses we tested for clonal analysis were 6.5 · 10^−12^, 3.25 · 10^−12^ and 6.5 · 8.25^−13^ U per embryo, refereed in the results sections as 1, 1/2 and 1/4, respectively.

### Determination of the injection site in the nascent mesoderm

We classified injections to the nascent mesoderm into two categories: proximal and distal. For that, we divided the proximal-to-distal length of the nascent mesoderm into two compartments of equal length. Then, we proceeded with the injection in either one or the other. As embryos in different stages present different lengths of the primitive streak/nascent mesoderm, these compartments were specific to every embryo. For example, an embryo in middle streak (MS) stage would present both compartments in the top half of the embryo whereas in an early bud (EB) stage embryo, they would occupy its whole length.

Then the angle of injection was selected by the manipulator. The more parallel to the surface of the Petri dish the needle lays, the closer this angle would be to 0°. In our case, the needle was set as parallel to the dish as possible, which corresponded to ∼5°. Then, injections were roughly aimed at the embryo mid-plane (primitive streak axis) by focusing the larger (centre) embryo slice.

### Immunohistochemistry

TAT-Cre recombinase-microinjected embryos were cultured for variable times and then removed from culture, PBS-washed and fixed in a 4% solution of paraformaldehyde (PFA) in PBS for 3-4 h at 4°C. Immunofluorescence was performed as follows: after washing with PBS twice, embryos were permeabilised with a 0.3% Triton X-100 solution in PBS for 30 min at room temperature. Blocking was carried out with bovine serum albumin 0.5% in PBS for at least 3 h at 4°C. Primary antibody incubation was performed overnight. Primary antibodies used were: anti-Mycn (1:100; NMYC-1, Santa Cruz Biotechnology, sc-80546), anti-phospho-Histone H3 (1:100; Millipore, 06-570), anti-M20 (1:100; anti-MF-20-mouse, Developmental Studies Hybridoma Bank) and anti-6xHis-tag (1:500; anti-polyHistidine-Peroxidase antibody, mouse monoclonal, Sigma-Aldrich, A7058). Primary antibody washing was performed in a 0.1% Triton X-100-PBS solution for at least 5 h at 4°C. Secondary antibody incubation was also performed overnight at 4°C. The secondary antibodies used were: Alexa Fluor 647 goat anti-mouse (1:500; Life Technologies, A31571), AlexaFluor 594 goat anti-rabbit (1:500; Life Technologies, A11037). For anti-6xHis-tag staining, embryos were washed in PBS for 2 days at 4°C and then incubated for 5 min with TSA Cyanine 5 at room temperature (Akoya Biosciences, NEL705A001). All embryos were stained for nuclei with DAPI diluted in PBS. The embryos were clarified in crescent dilution of glycerol in PBS (25%, 50% and 75%) until analysis was performed by confocal microscopy.

### Confocal imaging

Whole embryos mounted on a Leica TCS SP5 confocal microscope were imaged using 405, 488, 561 and 633 nm wavelengths and 10×/0.4 dry and 20×/0.75 dry objectives. Images were then analysed using Fiji-ImageJ (National Institutes of Health).

### Two-photon live imaging

Live imaging was performed as previously described (Sendra et al., 2022). Briefly, mouse embryos were harvested and dissected in pre-equilibrated dissection medium: DMEM supplemented with 10% foetal bovine serum, 25 mM HEPES-NaOH (pH 7.2), penicillin (50 g/ml) and streptomycin (50 g/ml). Embryos from E6.5 to E7.5 were cultured in culture medium: 50% Janvier Labs Rat Serum Sprague Dawley RjHan SD male only and 50% DMEM FluoroBrite (Thermo Fisher Scientific, A1896701). Temperature was set to 37°C, 7% CO_2_ concentration in a Zeiss LSM780. The objective lens used was a 20× (NA=1) dipping objective. A MaiTai laser line at 980 nm was used for two-channel two-photon imaging. Acquisition was carried out using Zen software (Zeiss). Output power was 250 mW, pixel dwell time 14.8 s, line averaging of two and image dimension of 610×610 m (512×512 pixels). To image up to five embryos simultaneously, we cyclically moved from one embryo to another by adjusting the frame and the *z*-stack every 15 min (imaging each embryo every ∼40-75 min). This was carried out for up to 8 h while the user was present. Then, one embryo was left for overnight imaging (at 10-15 min time frames) while the rest were cultured along in the same chamber.

### Clonal analysis

For clonal analysis, we considered ‘groups of labelled cells’ (Tomato or GFP) derived from single injections. The contribution of these labelled cells to each anatomical location was evaluated by counting DAPI nuclei within Tdtomato+ or GFP+ cells. Anatomical locations were identified using morphological features (Kaufman and Navaratnam, 1981) in the DAPI channel. Groups of labelled cells containing both Tomato+ and GFP+ cells were annotated as ‘bicolour’. The polyclonality of monocolour groups in the Cre recombinase-microinjected embryo collection was estimated using the frequency of bicolour groups as previously described (Lioux et al., 2020; Padrón-Barthe et al., 2014). This method is based in the fact that the frequency of bicolour events in a collection of samples is directly proportional to its polyclonality (Fig. S5A). This allows the calculation of the probability of finding groups of cells labelled with one reporter (monocolour) that originate from multiple progenitors (polyclonal) using the formula in Fig. S7F. For that, we first estimated the relative Tomato and GFP recombination frequency: RERT^+/−^;*ROSA26R^CAG-Tdtomato+/+^* mice were crossed with *ROSA26R^CAG-GFP+/+^* mice. Reporter recombination was induced by administering 0.04 mg/g of 4-OH tamoxifen intraperitoneally to pregnant females on day E7 (Fig. S5). A day later, the embryos were harvested and the relative efficiency of recombination was calculated by manually counting GFP and Tdtomato cells over total DAPI nuclei in confocal optical sections using the ImageJ Cell Counter plugin.

### Confocal image analysis

Once acquired, the images were opened as stacks of optical planes and saved in .tiff format. For fate mapping injections in the pSHF, we used the DAPI and MF20 immunostaining channels to identify the different heart chambers at E9.5. Next, we examined the GFP and Tdtomato channels to assess the contribution of the recombined cells to each of the anatomical locations.

For the measurement of proliferation rates in Tdtomato+and Tdtomato− heart cells, the number of Ph3-positive nuclei was counted in the Tomato+ and Tomato− regions. The Ph3 counts were then normalised to the total area of Tdtomato+ or Tdtomato− cells, which was measured using a custom Fiji macro. In brief, the user sets manually the correct threshold that selects the Tdtomato+ area and then the macro measures such area by applying a binary mask to the image. To test whether there was a difference in Ph3 counts between Tdtomato+ and Tdtomato− domains, we used the unpaired two-tailed *t*-test for two related samples. This is a test of the null hypothesis that two related or repeated samples have identical averages (expected) values.

## Supplementary Material

10.1242/develop.201206_sup1 Supplementary information
