# Supplementary material for: Cre recombinase microinjection for single-cell tracing and localised gene targeting
Source: Development. 2023 Feb 3;150(3):dev201206. doi: 10.1242/dev.201206 (PMC10110498; doi:10.1242/dev.201206)
Supplement: Supplementary information [file develop-150-201206-s1.pdf]

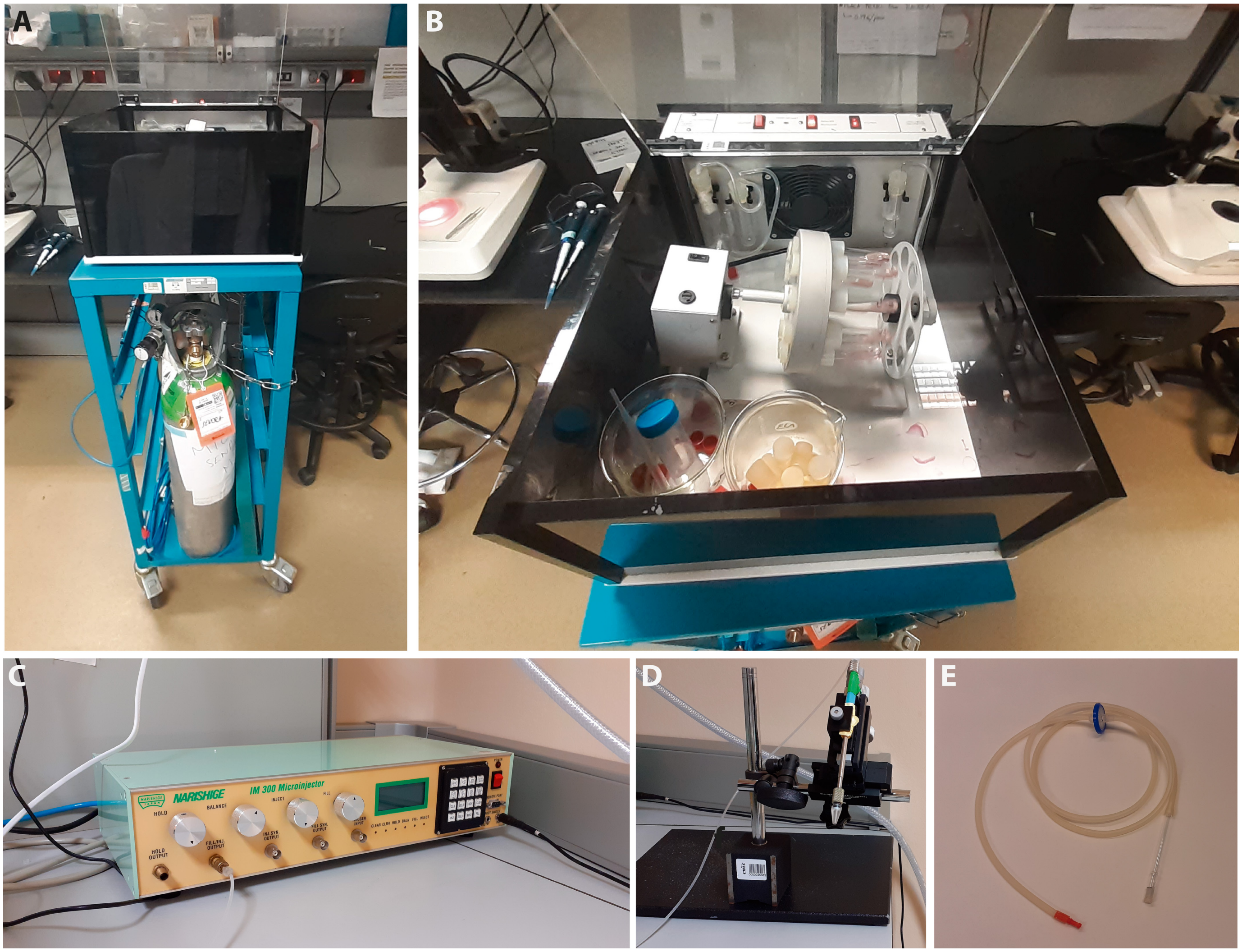

Fig. S1. Setting for manual microinjection in E7.5 to E8.5 mouse embryos. (A) and (B), 'Roller bottle' culture system. (C), Pneumatic microinjector. (D), Manual manipulator to aim the glass capillary at the desired injection site. (E), Glass capillary connected to a silicone tube with a mouthpiece.

Development • Supplementary information

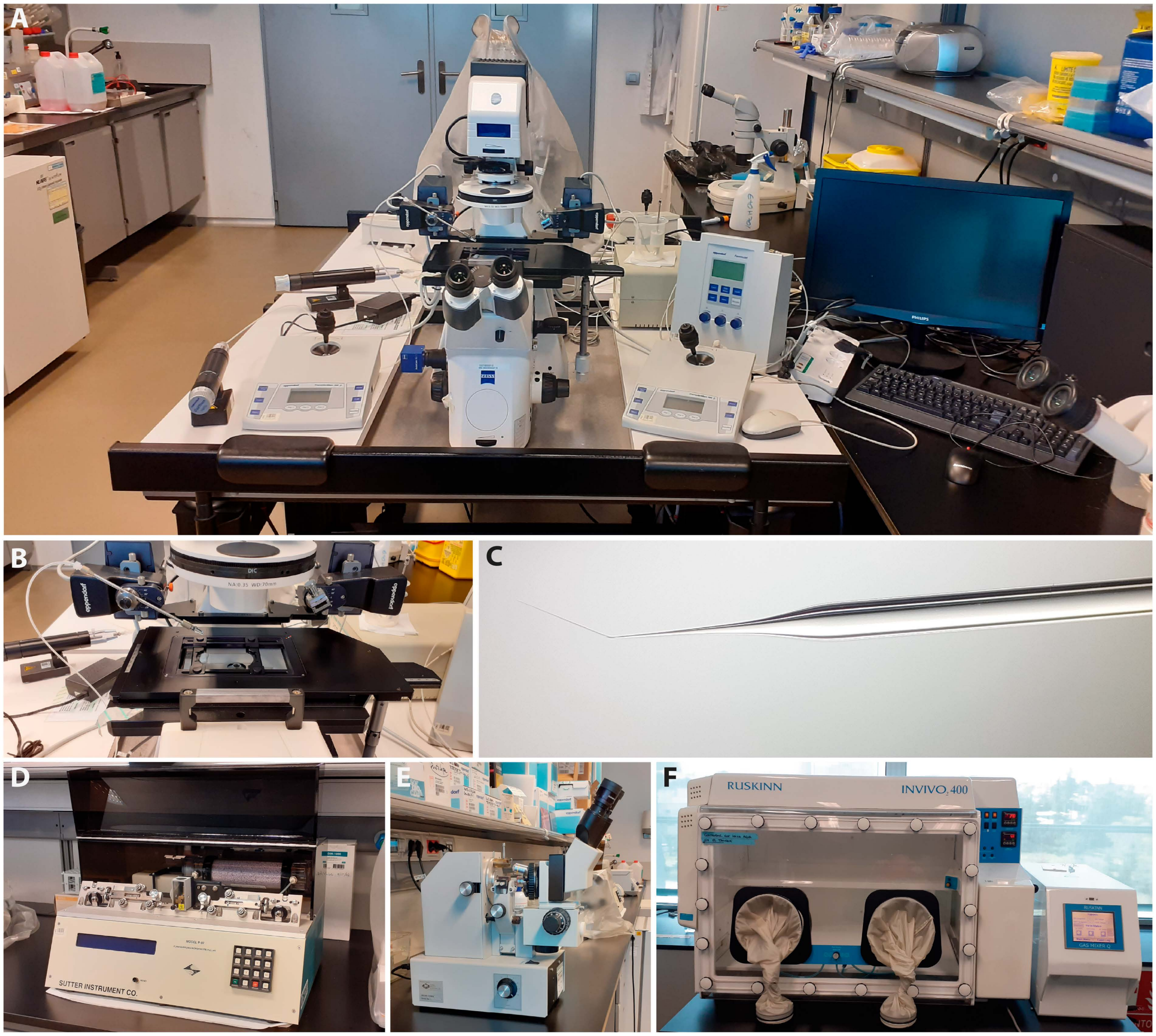

Fig. S2. Setting for micromanipulator–mediated microinjection in E6.5 to E7.25 mouse embryos. (A) and (B) Micromanipulation station. (C), Pulled glass capillaries for low–dose TAT–Cre microinjection. (D), Glass capillary puller. (E), Stereomicroscope for embryo selection. (F), Temperature and gas adjustable incubator.

Development • Supplementary information

A Summary of TAT–Cre microinjected and live–imaged embryos

●● Cells tracked for panel D

Embryo _ ID	Dose	Injection aim	Cell location	Number of cells	Cells tracked for panel D
e001	1/2	mesoderm	mesoderm	1 GFP	● (red)
e004	1/2	mesoderm	mesoderm	1 Tdtomato	● (red)
e002	1/2	ectoderm	no fluorescence	Ø	
e003	1/2	ectoderm	no fluorescence	Ø	
e005	1/2	ectoderm	no fluorescence	Ø	
e006	1	ectoderm	ectoderm	1 GFP	● (blue)
e007	1	mesoderm	mixed	1 Tdtomato and 1 GFP	● (red) ● (blue)
e008	1	mesoderm	mesoderm	1 Tdtomato and 1 GFP	● (red)
e009	1	mesoderm	mesoderm	2 Tdtomato	
e010	1	ectoderm (amniotic cavity)	mixed	>2 Tdtomato and 2 GFP	

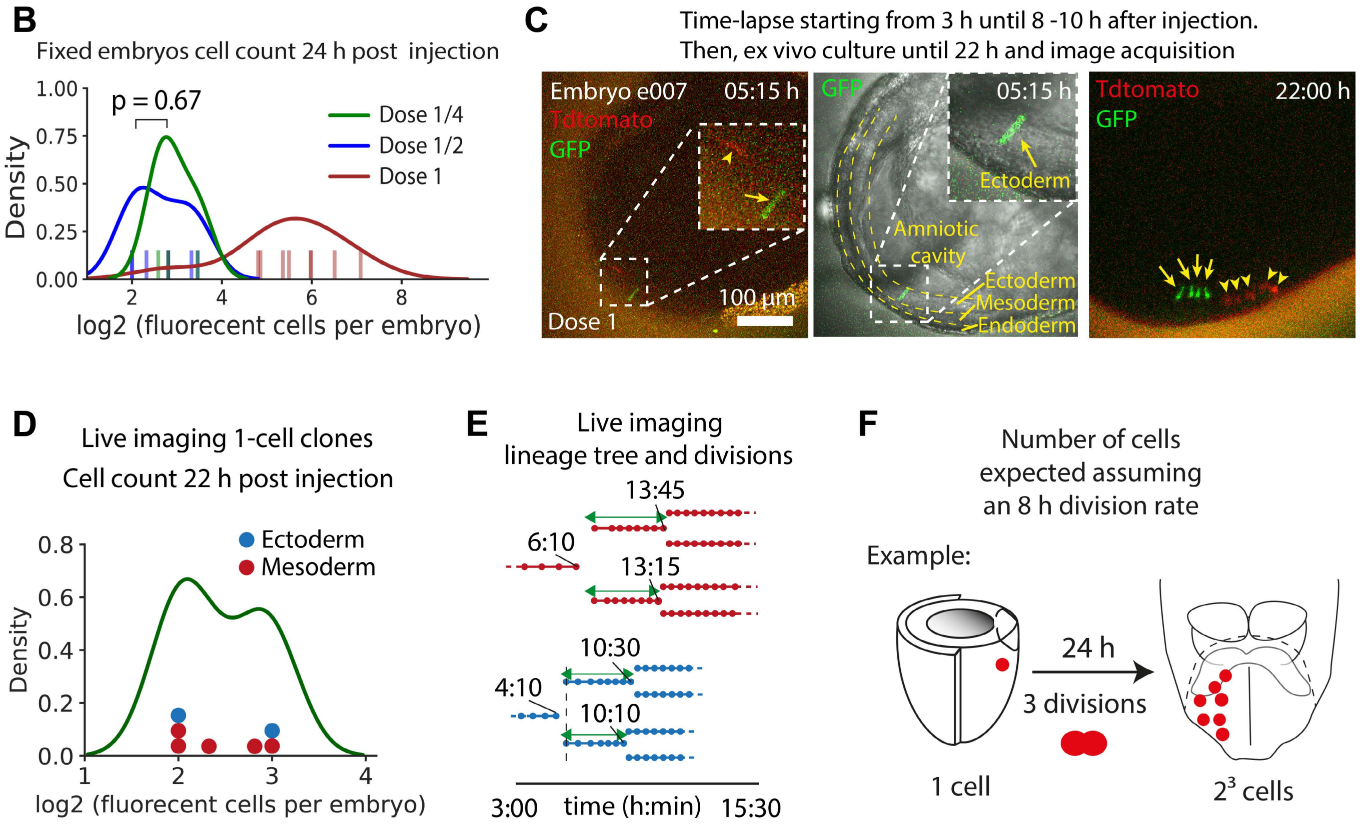

Development • Supplementary information

Fig. S3. Live imaging analysis of cell division and expected cell numbers for TAT–Cre generated clones. (A), Summary of microinjected live–imaged embryos.(B), Kernel density estimate revealing the distribution of the number of cells per embryo in the three different doses, and rug plots at the bottom showing cell counts for individual embryos (n = 18 embryos from 6 litters, a two-sample Kolmogorov-Smirnov test for goodness of fit was performed to compare the distributions of dose $1/2$ and dose $1/4$ samples). (C), Initial time point of an embryo showing two individual cells and their progeny after *ex vivo* culture. Yellow arrows point to ectodermal cells while arrowheads to mesodermal cells. Time is reported as hours:minutes. (D), Kernel density estimate revealing the distribution of the number of cells in live–imaged *bona ide* clones examined 22 h after culture. Individual counts per cell are represented as dots at the bottom (n = 7 cells from 5 embryos). Embryos e009 and e010 from A were not included because identification of single cell progenies in these embryos was difficult (two or more recombination events with the same reporters). (E), Reconstructed lineage trees from live imaging cell tracks. Each dot represents approximately the time of each 3D stack acquisition. The last time stamps before division was observed are annotated on top of the branches. The time between two cell divisions is highlighted with a green double arrow line. The plot is not scaled (n = 4 divisions, 2 cells from 2 embryos). (F), Expected cell number rationale.

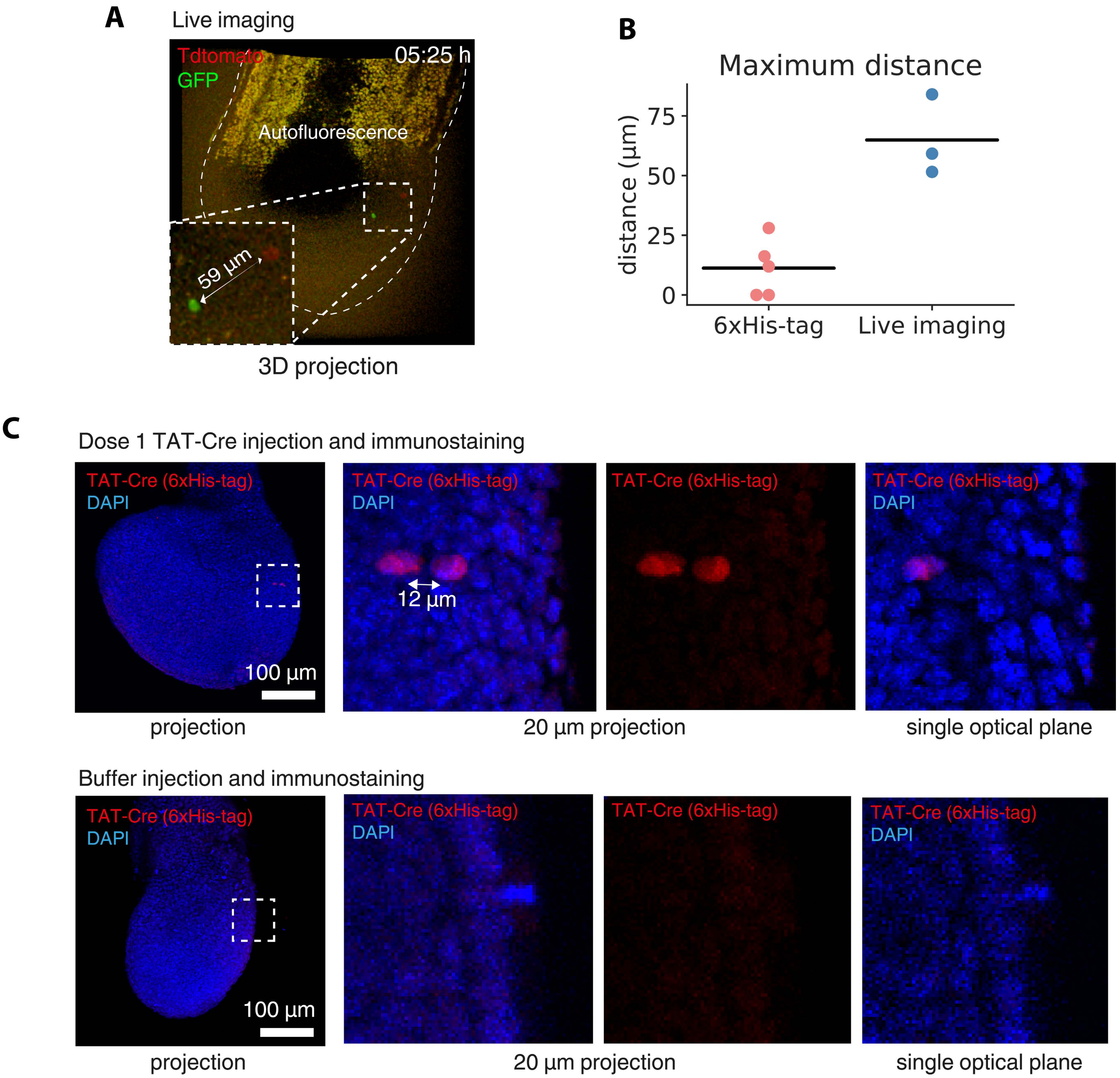

Fig. S4. Spatial range of recombination for TAT–Cre microinjection. (A), Distance between cells that recombine from the same injection (n = 3 pairs of cells from 3 embryos). (B), Dot plots showing the maximum distance between cells positive for 6xHis–tag staining or recombined in Live imaging data. Means are shown as horizontal lines. (C), Immunostaining for the polyhistidine tag (6xHis–tag) reveals ectoderm cells with TAT–Cre signal after injection, which is absent in negative control embryos injected with buffer (n = 5 TAT–Cre injected, 3 buffer injected).

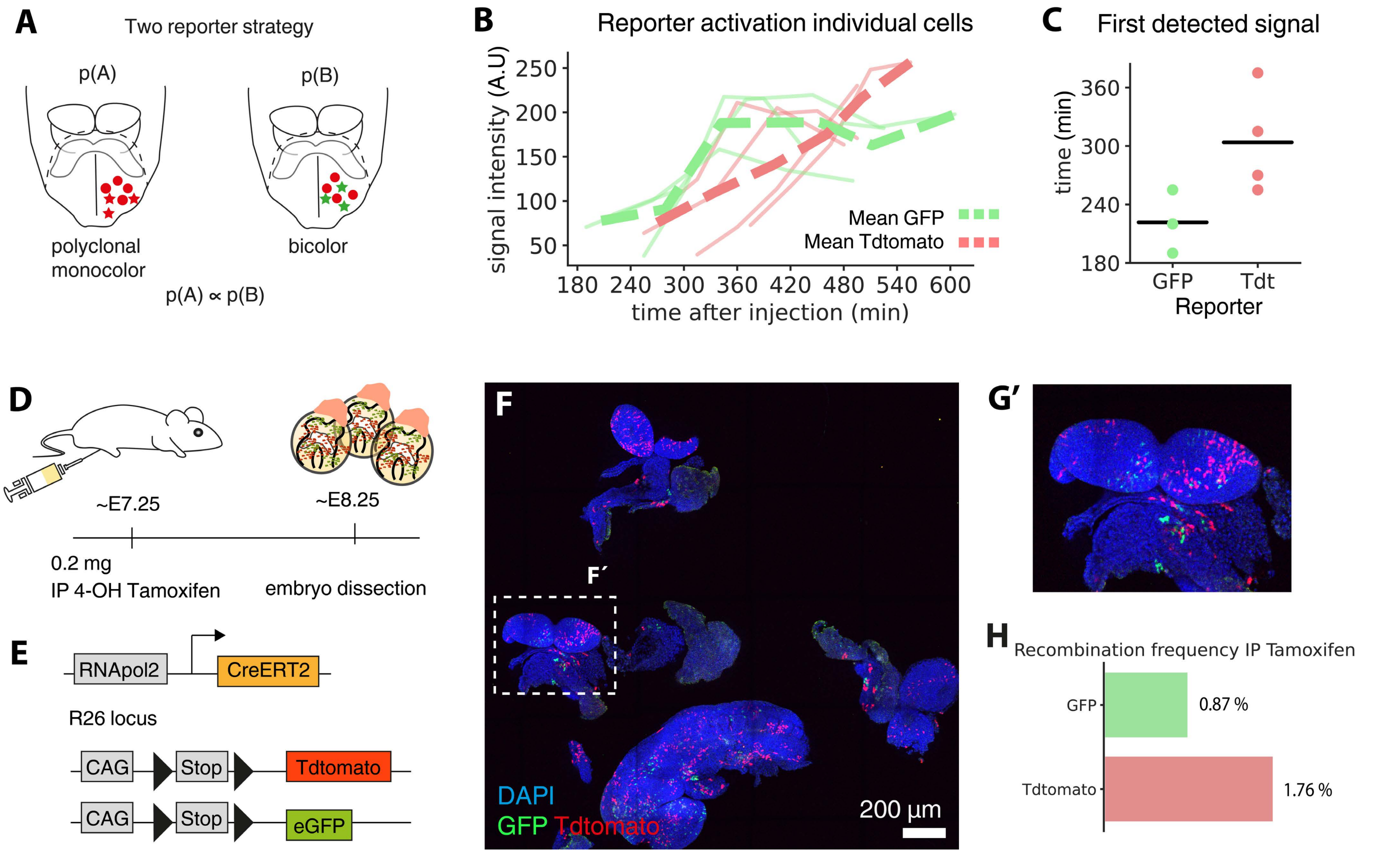

Fig. S5. Timing of detection and recombination efficiency of Tdtomato and eGFP reporters. (A), Two reporter strategy rationale. The probabilities of two inde-pendent recombination events giving rise to same or different reporter combinations are proportional. (B), Line plots show the reporter intensity in tracked cells. Solid lines correspond to individual cells while dotted lines represent the average for each reporter (n = 7 cells from 5 embryos). (C), Time where reporters are first detected (n = 7 cells from 5 embryos). (D), Intraperitoneal (IP) tamoxifen microinjection setup to calculate relative Tdtomato and eGFP expression frequency. (E), Embryo's genetic background for tamoxifen–inducible reporter expression. (F), Confocal intensity projection of IP tamoxifen–induced fluorescent embryos. (F') Inset of (F). (G), Recombination frequency of GFP and Tdtomato.

Development • Supplementary information

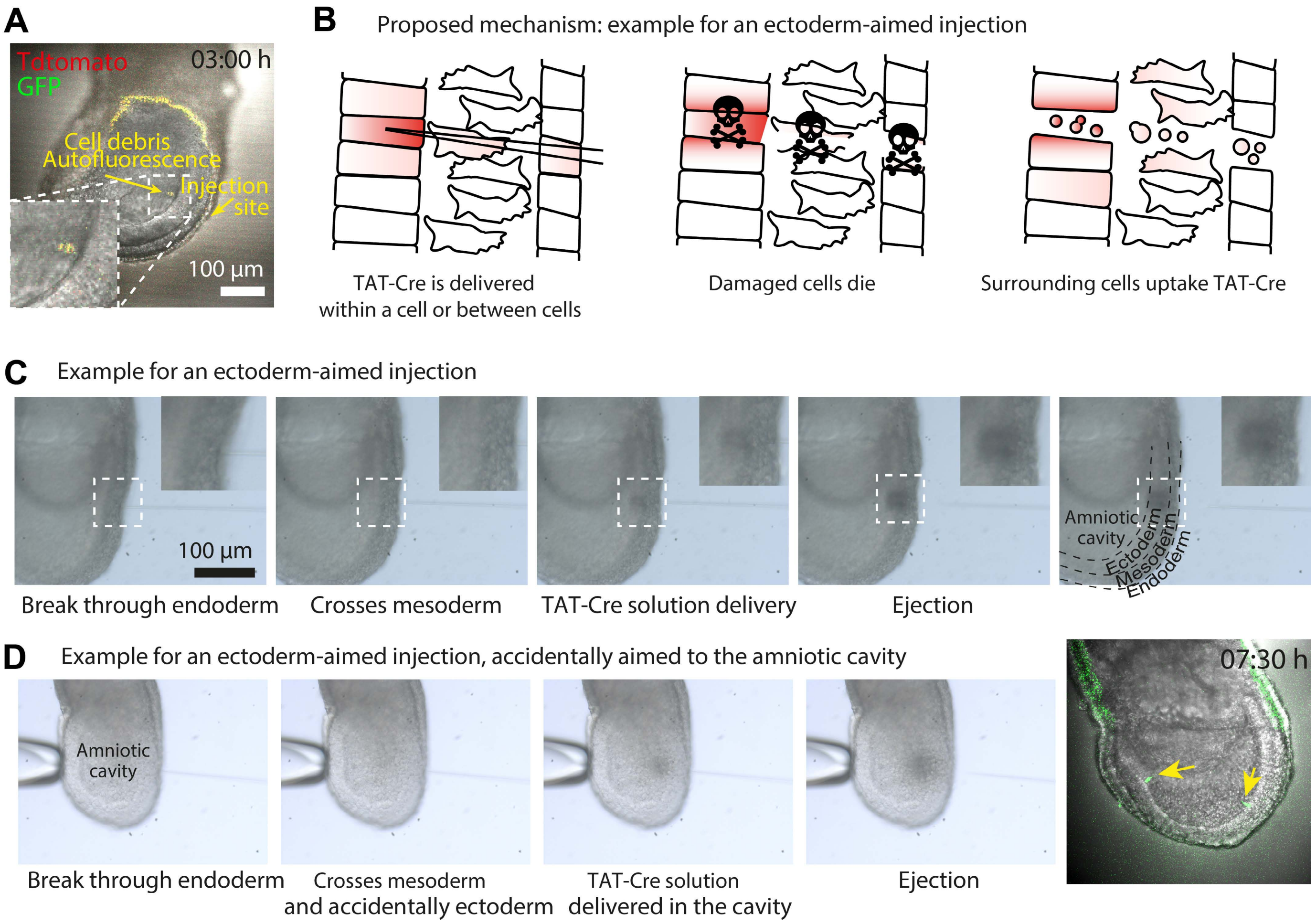

Fig. S6. Cell death and off–target recombination using TAT–Cre microinjection. (A), Time stamp showing cell debris in the amniotic cavity of a microinjected embryo. (B), Proposed mechanism for TAT–Cre targeting. (C), Time series of an embryo microinjected in the ectoderm. (D), Time series of an embryo accidentally microinjected in the amniotic cavity. Yellow arrows point at GFP recombined cells.

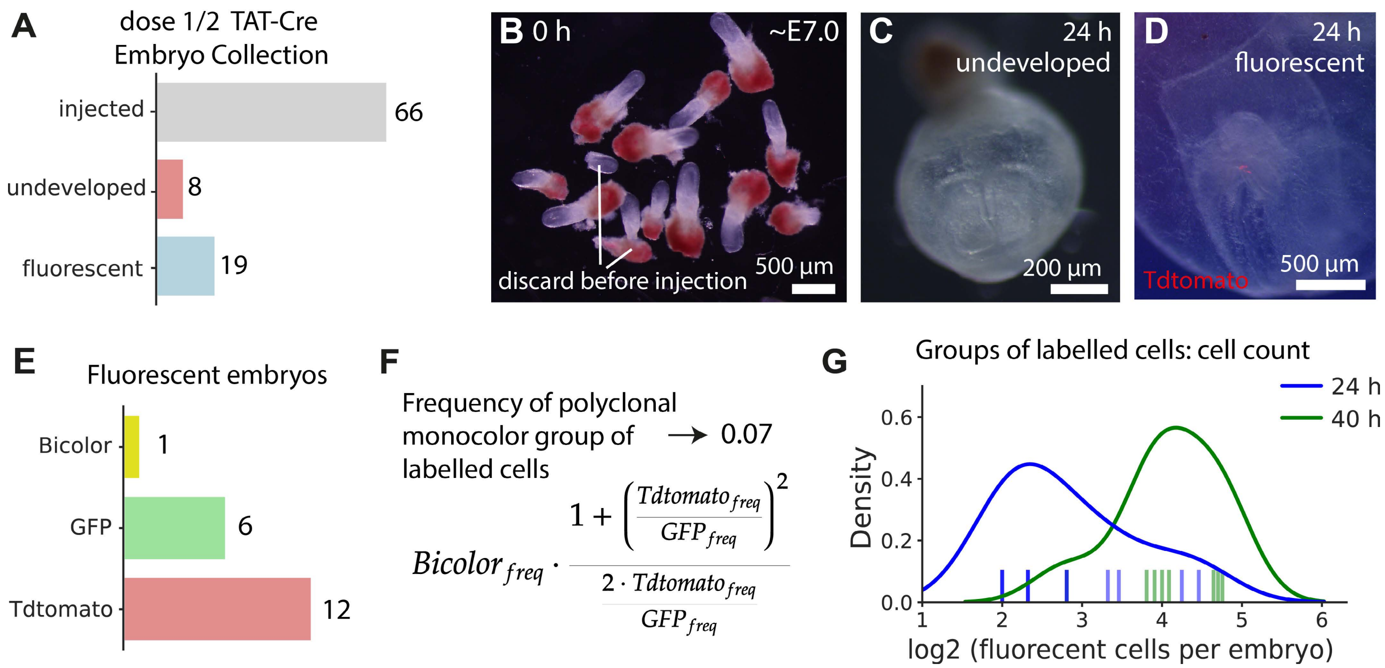

Fig. S7. Quality control of early heart progenitors prospective clonal analysis using TAT–Cre. (A), Bar graph shows the number of injected, undeveloped and fluorescent embryos obtained after culture (n = 66 embryos from 8 litters). (B), Dissected E7.0 embryos before TAT–Cre microinjection. (C), Undeveloped embryo after 24 h culture. (D), Fluorescent Tdtomato group of labelled cells in a microinjected embryo 24 h after culture. (E), Number of embryos with a GFP, Tdtomato, or bicolour. (F), Formula to estimate the probability of getting a monocolour group of labelled cells arising from recombination in two different cells.(G), Kernel density estimate revealing the distribution of the number of cells per embryo, and rug plots at the bottom showing the cell counts of individual embryos.

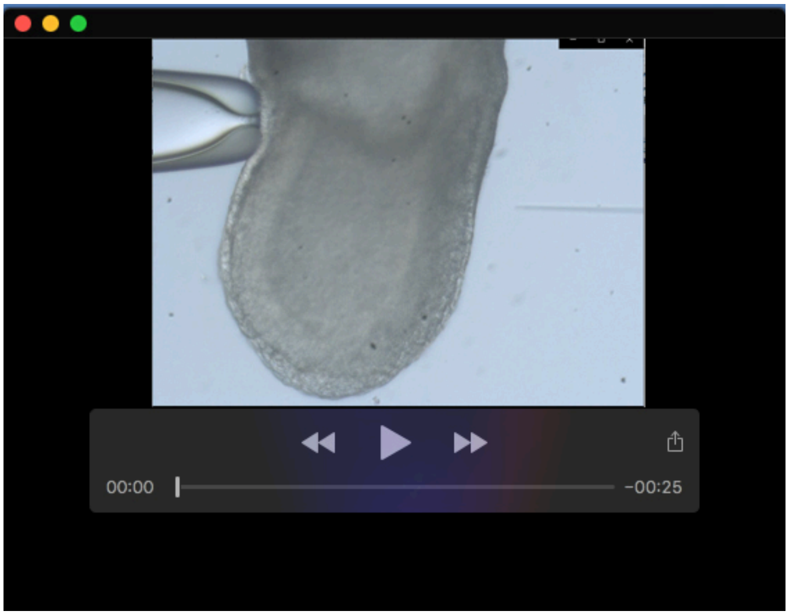

Movie 1. TAT–Cre microinjection of a ∼E7.25 mouse embryo in the mesoderm using a zygotic microinjector.

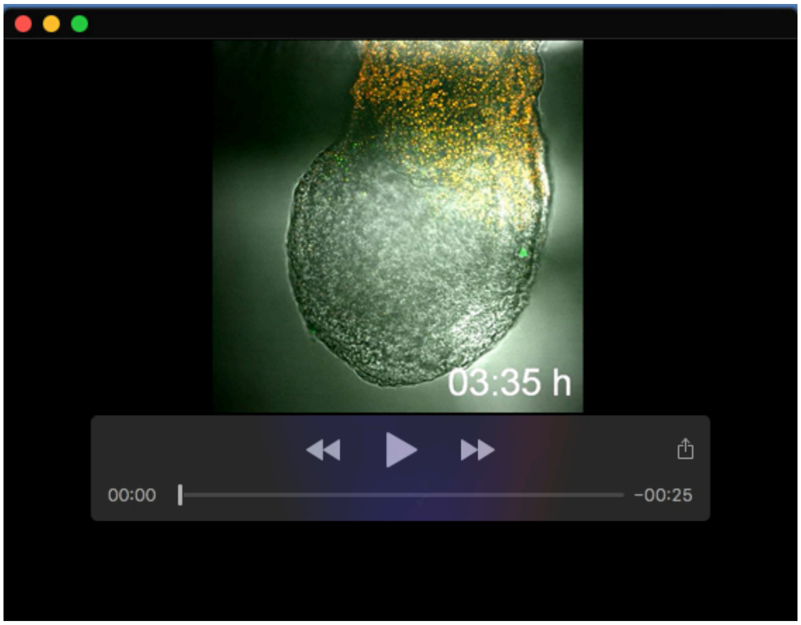

Movie 2. Live imaging of embryo 001, microinjected with dose 1/2 TAT–cre in the mesoderm.

Development • Supplementary information
