# Supplementary material for: Cre recombinase microinjection for single-cell tracing and localised gene targeting
Source: Development. 2023 Feb 3;150(3):dev201206. doi: 10.1242/dev.201206 (PMC10110498; doi:10.1242/dev.201206)
Supplement: Supplementary file 1 [file develop-150-201206_review_history.pdf]

Cre recombinase microinjection for single-cell tracing and localised gene targeting

Miquel Sendra, Juan de Dios Hourcade, Susana Temiño, Antonio J. Sarabia, Oscar H. Ocaña, Jorge N. Domínguez and Miguel Torres
DOI: 10.1242/dev.201206

Editor: Benoit Bruneau

Review timeline

Original submission:	16 August 2022
Editorial decision:	23 September 2022
First revision received:	22 November 2022
Editorial decision:	9 December 2022
Second revision received:	13 December 2022
Accepted:	17 December 2022

Original submission

First decision letter

MS ID#: DEVELOP/2022/201206

MS TITLE: Cre recombinase microinjection for single-cell tracing and localised gene targeting

AUTHORS: Miquel Sendra, Juan de Dios Hourcade Bueno, Susana Temiño, Antonio José Sarabia, Oscar Horacio Ocaña, Jorge Nicolás Domínguez, and Miguel Torres

I have now received all the referees reports on the above manuscript, and have reached a decision. The referees' comments are appended below, or you can access them online: please go to BenchPress and click on the 'Manuscripts with Decisions' queue in the Author Area.

The overall evaluation is positive and we would like to publish a revised manuscript in Development, provided that the referees' comments can be satisfactorily addressed. Please attend to all of the reviewers' comments in your revised manuscript and detail them in your point-by-point response. If you do not agree with any of their criticisms or suggestions explain clearly why this is so. If it would be helpful, you are welcome to contact us to discuss your revision in greater detail. Please send us a point-by-point response indicating your plans for addressing the referee’s comments, and we will look over this and provide further guidance.

Reviewer 1

Advance summary and potential significance to field

The paper describes a method for lineage tracing and local gene ablation in post-implantation mouse embryos. The authors use microinjection of cell-permeant Cre recombinase through either pneumatic microinjection or a zygotic microinjection setup, depending on the embryo stage. They provide detailed protocols for the injection method, the choice of dosage, the culture conditions, and the analysis of the injected embryos. They show several examples highlighting the applicability of the method for lineage tracing of cardiac cell populations at different stages, as well as for localised gene deletion.

© 2023. Published by The Company of Biologists under the terms of the Creative Commons Attribution License (https://creativecommons.org/licenses/by/4.0/).

Single cell labelling in post-implantation mouse embryos is technically challenging, particularly in the mesoderm layer and its derivatives, so the method elegantly meets a need. It is clearly written and illustrated, so that it should be reproducible by any laboratory.

Comments for the author

Minor comment:
I may have missed it, bu could not find a reference for the TAT-Cre. Have you tested different commercially available ones, or do you produce your own? A description of the source and dilution buffer/method would be useful.

Reviewer 2

Advance summary and potential significance to field

In this study Sendra and colleagues describe a Cre-based microinjection approach to investigate lineage and localized gene regulation during gastrulation and early organogenesis focusing on cardiac development. The authors show that this approach can be used to perform prospective lineage analysis and propose that it enables them to assess the single-cell clonal dynamics of epiblast/early mesoderm progenitors. The authors then use the same technique to perform localized gene knock-down and highlight a cell-autonomous role for Mycn in maintaining cardiac proliferation. The authors should be commended on their skillful ability to handle, microinject and culture murine embryos. Overall this paper provides an innovative way to look at localized gene modifications and potentially illustrates an advance in techniques available to perform prospective lineage analysis although further validation of this single-cell clonal approach is required.

Comments for the author

Major;
The authors report that this technique provides a method to conduct single-cell prospective lineage labeling, however from the data provided it is not clear what is being labeled following microinjection and conclusions regarding the single-cell labeling are based on estimations/assumptions.

To gain accurate biological insight form lineage based approaches it is critical to understand the number and types of cells initially being labeled. The current manuscript does not definitely address this and whilst it corroborates previous findings using other lineage analysis approaches it does not provide enough technical validation to make definite conclusions in regards to single-cell clonal analysis. For example in Figure 3 monocolour clones span anatomically diverse tissue types as well as different germ layers (Cluster 8 = ExM, PsM and End; Cluster 9 = PsM, End, Ecto). Does this represent labeling of a single pluripotent epiblast cell, an early Mesendoderm progenitor or has Cre labeling occurred in multiple cells during injection? Given the authors skilled ability to handle and manipulate embryos, data showing what is being labelled/exposed to CRE shortly after microinjection should be provided. As a control experiment could a dye be injected alongside the TAT-Cre or combining this approach with a barcode lineage mouse model, such as the Polylox system, would provide validation.

Data should be provided to support the cell division rate when using this experimental setup. "We expect cells to divide around 3 times in 24h" (line 179), given this assumption is reported to "confirm" single cell labeling (175), it should be validated. Does this hold true for microinjected cultured cells/embryo

Technique and Resource reports should be formatted to combine results and discussion sections. Combining these sections would generate more space for the details and data required to validate assumptions, clarify analytical approaches and provide alternative interpretations of the data presented.

© 2023. Published by The Company of Biologists under the terms of the Creative Commons Attribution License (https://creativecommons.org/licenses/by/4.0/).

Minor;

What is a low probability of producing a polyclonal cluster (line 184)? How is this determined if there are no bicolour clones detected Figure 2?

What is a coherent cluster (line 189)? How are clusters defined? Padron-Barthe et al. 2014 states that optimal threshold distance depends on each experimental setting. How do clusters and clone relate?

Typo in figure 2 – Policlonal

Data in figure 3 seems to suggests 20 clones, how does this relate to the 19 embryos/clusters described in the text and legend?

In which region of the PS were embryos injected for experiments in 3A and how was this controlled given the different stages injected. How were proximal/distal regions determined given the variations in embryo size and stage. Injections in Figure 3B and H both in proximal half but are very different in regards to position in the streak whilst panels B and F are in opposing halves (distal and proximal) but are probably more closely related in terms of position in the streak.

Line 198 - Our collection of clones induced in the posterior epiblast and the primitive streak contributed to anterior mesodermal locations. What is being labeled? Is it a single cell or a region of cells - this will be crucial for future interpretation of results.

I would debate whether this technique "represents a simple protocol which does not require specific technical skills", I feel the authors are being modest and should be complemented on their ability to both manipulate and culture gastrulating murine embryos.

<u>Reviewer 3</u>

Advance summary and potential significance to field

Reconstructions of cell lineages is essential to understand morphogenesis mechanisms. This has remained challenging in the mouse due to inaccessibility of the embryo in utero. Genetic tools for cell labelling and single cell RNA sequencing have provided insights into mouse cell lineages. Sendra et al have now developed a new approach for prospective cell labelling in the mouse embryo, which provides easier and longer clonal labelling compared to previous approaches. This is an excellent report for the Techniques and Resources section, which describes and controls carefully the new approach, by reproducing previous results. It is well referenced, exemplifies applications for fate mapping, clonal analyses and clonal genetic perturbations.

Comments for the author

Major comments
I have two pending questions on the approach.

1-A point which is currently not covered by the report is the definition of initial labelled cells in space and time. Is the injection between cells or within a cell and does it cause cell lethality ? How far from the injection site can the TAT-Cre diffuse or transfect cells ? How long does it take from the time of injection to the time of labelling ? Cre mediated recombination is known to cause a delay in cell targeting and hence can question whether “primitive streak progenitors” are labelled or their descendants. Similarly if the Cre diffuses, is it nascent mesoderm or epiblast cells which are targeted ?
For fate mapping, a key parameter is to record the site of injection. Whereas the proximal-distal position may be quantified from the picture of the needle, the radial position is more difficult to assess. K. Lawson was using a second injection in the extra-embryonic visceral endoderm, that grows coherently and thus provides a signature in the yolk sac of the radial position. What is the strategy here ?

© 2023. Published by The Company of Biologists under the terms of the Creative Commons Attribution License (https://creativecommons.org/licenses/by/4.0/).

2-It is unclear why monoclonal clusters are considered to control clonality (Fig. 2), whereas labelling of different tissues in Fig. 3 may not be as a continuous cluster. Considering single clusters is an understatement of the clone, given the existence of clone dispersion, as for example for cardiac cells before heart tube formation. How is the distribution of the total number of fluorescent cells per embryo (like Fig. 2F) ? And how is the calculation of clonality (Fig. S3F) for the total number of fluorescent cells per embryo ? Does this vary with the duration of culture (and potential duration of TAT-Cre activity) ?

Minor comments
-please add the source of TAT-Cre : home-made or commercial ?
-Fig1E has red cells in the OFT : why is this not shown in Fig1B and not mentioned in the text ?
-In Fig3A : please specify the injection site of each clone and highlight clones considered in Fig 3D versus E
-annotate Fig.3 C, G, I to show tissues
-line 202-205 clarify your results compared to cited refs
-line 210 : Tyser et al and Zhang et al don't show the primitive streak origin
-typo "policlonal" and "fluorecent" Fig 2G-H, "early streak (MS)" Fig 3A, "pooled" and "pooler" FigS2

First revision

Author response to reviewers' comments

Reviewer 1 Comments for the Author:

Minor comment:
I may have missed it, bu could not find a reference for the TAT-Cre. Have you tested different commercially available ones, or do you produce your own? A description of the source and dilution buffer/method would be useful.

We have now added the reference for the TAT-cre and dilution buffer in the Methods section.

Reviewer 2 Comments for the Author:
Major;
The authors report that this technique provides a method to conduct single-cell prospective lineage labeling, however from the data provided it is not clear what is being labeled following microinjection and conclusions regarding the single-cell labeling are based on estimations/assumptions.

To gain accurate biological insight form lineage based approaches it is critical to understand the number and types of cells initially being labeled. The current manuscript does not definitely address this and whilst it corroborates previous findings using other lineage analysis approaches it does not provide enough technical validation to make definite conclusions in regards to single-cell clonal analysis. For example in Figure 3 monocolour clones span anatomically diverse tissue types as well as different germ layers (Cluster 8 = ExM, PsM and End; Cluster 9 = PsM, End, Ecto). Does this represent labeling of a single pluripotent epiblast cell, an early Mesendoderm progenitor or has Cre labeling occurred in multiple cells during injection? Given the authors skilled ability to handle and manipulate embryos, data showing what is being labelled/exposed to CRE shortly after microinjection should be provided. As a control experiment could a dye be injected alongside the TAT-Cre or combining this approach with a barcode lineage mouse model, such as the Polylox system, would provide validation.

We agree with the reviewer that our manuscript would benefit from direct evidence of what is being labelled after injection. For this, we performed two new experiments: live imaging and TAT-Cre (6xHis-Tag) immunostaining of TAT-Cre microinjected embryos.

© 2023. Published by The Company of Biologists under the terms of the Creative Commons Attribution License (https://creativecommons.org/licenses/by/4.0/).

Using live imaging, we back--tracked recombined cells, which allowed us to analyze whether single or multiple cells recombine using different TAT-Cre doses. Then, the number of cells / number of divisions obtained in *bona fide* live imaging clones were compared to those in our collection of fixed embryos. These results are now included in Figure 2 and Figure S3 and Results and Discussion section “Titration of TAT-Cre recombinase for clonal analysis” paragraphs 3-6.

To understand better what is being labelled/exposed to CRE shortly after microinjection, we tried injecting the lipophilic DiR alongside TAT-Cre but the DiR precipitated and blocked the needle. Furthermore, co-injected dyes will diffuse at their own dynamics, which would not necessarily mimic TAT-Cre diffusion. Instead, we immunostained the polyhistidine tail (6xHis-tag) present in TAT-Cre, to directly visualize the injected protein. We also measured the distance between multiple cells recombining in the same embryo in live-imaging, which allowed us to estimate the effective range of Cre-induced recombination. The results show that recombinations occur within a narrow range from the injection site. These results are now included in figure S4 and Results and Discussion section “Spatial and temporal resolution of TAT-Cre recombination” paragraphs 2-4.

As for the question of whether cluster 8 and 9 in figure 3 correspond to multiple recombinations, we discuss that they may have originated from recombination in two cells from different germ layers. This is included in the second to last paragraph of section “Prospective clonal analysis of nascent mesoderm progenitors”.

Data should be provided to support the cell division rate when using this experimental setup. "We expect cells to divide around 3 times in 24h" (line 179), given this assumption is reported to "confirm" single cell labeling (175), it should be validated. Does this hold true for microinjected cultured cells/embryo.

We now included cell numbers and cell division rates directly calculated using live imaging data. This is included in Results and Discussion section “Titration of TAT-Cre recombinase for clonal analysis” second to last paragraph. The results are compatible with cell divisions taking place every 7-8 h

Technique and Resource reports should be formatted to combine results and discussion sections. Combining these sections would generate more space for the details and data required to validate assumptions, clarify analytical approaches and provide alternative interpretations of the data presented.

We have now combined both sections in one. We also have subdivided the clonal analysis section in three parts, for clarity.

Minor;

What is a low probability of producing a polyclonal cluster (line 184)? How is this determined if there are no bicolour clones detected Figure 2?

In this experiment, we aimed to find a TAT-Cre dose that was low enough so that injections yielded infrequent recombination events. Although related to, recombination efficiency is not the same as the probability of producing polyclonal clusters. The way the manuscript was written led to confusion between these two concepts. We have now restructured the section “Titration of TAT-Cre recombinase for clonal analysis” so that labelling efficiency and polyclonality are addressed in different paragraphs.

What is a coherent cluster (line 189)? How are clusters defined? Padron-Barthe et al. 2014 states that optimal threshold distance depends on each experimental setting. How do clusters and clone relate?

We thank the reviewer for pointing out this mistake. In fact, in this study we do not use the coherence of the clusters to tell whether it contains one or several clones. Here, we use the whole embryo as a scoring unit, given that the recombination is triggered in a single anatomical location by a single injection. This means we do not look at the distribution of labelled cells to determine clusters, but take any fluorescent cell in each embryo as part of the same cluster.

© 2023. Published by The Company of Biologists under the terms of the Creative Commons Attribution License (https://creativecommons.org/licenses/by/4.0/).

Studying the coherence of clusters is only necessary when one needs to discriminate several groups of cells within the same embryo. For example, in Padron-Barthe et al. 2014, the recombination is induced randomly in the whole embryo by retrospective clonal analysis. Thus, several recombination events can be induced in distant parts of the embryo, yet one can tell them apart if setting a consistent distance threshold.

In our case, we do not rely on coherence. We calculate the probability that a dose ½ injection causes recombination of more than one cell, by counting the frequency of bicolor specimens in the collection, by counting the number of cells and by analyzing the live imaging data.

For these reasons, we removed the word "coherent" from the manuscript and corrected the Methods section:
"For clonal analysis, we considered "clusters" groups of cells (Tomato and/or GFP) derived from single injections."

Typo in figure 2 - Policlonal
We thank the reviewer for pointing out this typo. We have corrected it in the new version.

Data in figure 3 seems to suggests 20 clones, how does this relate to the 19 embryos/clusters described in the text and legend?
There is one bicolor cluster (which by definition contains 2 clones, a GFP recombination event and a Tdtomato recombination event). Then, the resulting number of clones increases by +1 respect to the number of clusters/fluorescent embryos.

In which region of the PS were embryos injected for experiments in 3A and how was this controlled given the different stages injected. How were proximal/distal regions determined given the variations in embryo size and stage. Injections in Figure 3B and H both in proximal half but are very different in regards to position in the streak whilst panels B and F are in opposing halves (distal and proximal) but are probably more closely related in terms of position in the streak.

We thank the reviewer for bringing up this topic. We agree that the determination of proximal/distal regions is not obvious. The primitive streak and resulting nascent mesoderm are a dynamic structure which extension changes rapidly in mouse embryos. We have now included a new methods section "Determination of the injection site in the nascent mesoderm."

Regarding injection sites showed in figure 3B, the image was annotated wrongly. As the reviewer pointed out, clone 4 was injected in the proximal region (see Figure 3A). We have corrected it.

Line 198 - Our collection of clones induced in the posterior epiblast and the primitive streak contributed to anterior mesodermal locations. What is being labeled? Is it a single cell or a region of cells - this will be crucial for future interpretation of results.

As demonstrated in the previous paragraph of that same section, these have a 93% probability of corresponding to single cells. In addition, the section "Titration of TAT-Cre recombinase for clonal analysis" now includes additional live-imaging experiments supporting that.

I would debate whether this technique "represents a simple protocol which does not require specific technical skills", I feel the authors are being modest and should be complemented on their ability to both manipulate and culture gastrulating murine embryos.

We agree with the reviewer that manipulating and culturing early post--implantation mouse embryos is not an easy task. It takes practice to achieve the dissecting skills required. What we intended to say here is that the injections are assisted by equipment that is commonly used in developmental biology laboratories, making the setup simpler. Yet the manipulation is complex.

We changed the last sentence of "Results and discussion" to clarify that, although the injections are assisted and relatively straightforward, the method still requires a user that is skilled in dissection.

© 2023. Published by The Company of Biologists under the terms of the Creative Commons Attribution License (https://creativecommons.org/licenses/by/4.0/).

Reviewer 3 Comments for the Author:
Major comments
I have two pending questions on the approach.

1-A point which is currently not covered by the report is the definition of initial labelled cells in space and time.
We thank the reviewer for bringing up this topic. We performed two new experiments: live imaging and TAT-Cre (6xHis-Tag) immunostaining of TAT-Cre microinjected embryos. The results are included in a new Results and Discussion section “Spatial and temporal resolution of TAT-Cre recombination”. We believe they clarify the definition of initial labelled cells in space and time and answer the reviewer’s specific questions:

- Is the injection between cells or within a cell and does it cause cell lethality ?
- We lack direct evidence to answer whether the injection occurs intracellularly or between cells. Associated to the injection site, we often observed cell debris, which suggests that the needle kills cells at the site of injection, while surviving surrounding cells may uptake TAT-Cre and recombine. We address this in the last paragraph of section “Spatial and temporal resolution of TAT-Cre recombination” (Figure S6A and B).

- How far from the injection site can the TAT-Cre diffuse or transfect cells ?
- We immunostained the polyhistidine tail (6xHis-tag) present in TAT-Cre after injection. Also, the distance between multiple cells recombining in the same embryo in live-imaging data allowed to estimate how far apart cells can be exposed to CRE. The results indicate that recombination takes place within a narrow range from the injection site. These results are now included in figure S4 and Results and Discussion section “Spatial and temporal resolution of TAT-Cre recombination” paragraphs 2 and 4.

- How long does it take from the time of injection to the time of labelling ?
- Between 2-3 and 7 h. To assess how long it takes from injection time to labelling time, we quantified the dynamics of fluorescence intensity in live imaging data. These results are now included in Figure S5B and C and paragraph 3 of Results and Discussion section “Spatial and temporal resolution of TAT-Cre recombination”.

- Cre mediated recombination is known to cause a delay in cell targeting and hence can question whether “primitive streak progenitors” are labelled or their descendants.
- We agree that this method does not offer enough temporal resolution to confirm that primitive streak progenitors are labelled. We have changed now “primitive streak” by “nascent mesoderm” in all of the manuscript’s instances.
- Similarly if the Cre diffuses, is it nascent mesoderm or epiblast cells which are targeted ?
- In the live-imaging experiments, we observed that aiming the injection at the mesoderm does not rule out the recombination of TAT-Cre in neighbouring ectodermal or endodermal cells. We now address this in the second to last paragraph in section “Prospective clonal analysis of nascent mesoderm progenitors”.

For fate mapping, a key parameter is to record the site of injection. Whereas the proximal- distal position may be quantified from the picture of the needle, the radial position is more difficult to assess. K. Lawson was using a second injection in the extra-embryonic visceral endoderm, that grows coherently and thus provides a signature in the yolk sac of the radial position. What is the strategy here ?

The embryo is set in an anterior to posterior position (with the sagittal plane perpendicular to the observer) and held with the pipette. Then the angle of injection is selected by the manipulator. The more parallel to the surface of the petry dish the needle lays, the closer this angle would be to 0. In our case, the needle was set as parallel to the dish as possible, which corresponded to approximately 5°. Then, injections were roughly aimed at the embryo’s midplane (primitive streak axis) by focusing the larger embryo’s slice. We considered this was accurate enough for the aim of this experiment, which was not to perform a fate map but to test TAT-Cre as a clonal analysis technique. However, to perform a detailed fate map, one would need to find a more accurate way to determine the radial position of the injection in respect to the embryo’s mid plane (such as K. Lawson’s second injection in the Extra-embryonic endoderm).

© 2023. Published by The Company of Biologists under the terms of the Creative Commons Attribution License (https://creativecommons.org/licenses/by/4.0/).

We have now included this information in Methods, section “Determination of the injection site in the nascent mesoderm.” last paragraph.

2-It is unclear why monoclonal clusters are considered to control clonality (Fig. 2), whereas labelling of different tissues in Fig. 3 may not be as a continuous cluster. Considering single clusters is an understatement of the clone, given the existence of clone dispersion, as for example for cardiac cells before heart tube formation. How is the distribution of the total number of fluorescent cells per embryo (like Fig. 2F) ? And how is the calculation of clonality (Fig. S3F) for the total number of fluorescent cells per embryo ? Does this vary with the duration of culture (and potential duration of TAT-Cre activity) ?
We thank the reviewer for pointing this out. In the new version of the manuscript, we made an effort to better explain the clonality criteria.

In this study we do not use the coherence of the clusters to tell whether it contains one or several clones. We use the whole embryo as a recombination unit, as the recombination is triggered in a single anatomical location by a single injection.

Studying the coherence of clusters is only necessary when one needs to discriminate if various groups of cells represent different clones within the same specimen. For example, in Padron-Barthe et al. 2014, the recombination is induced randomly in the whole embryo by retrospective clonal analysis. Thus, several recombination events can be induced in distant parts of the embryo, yet one can tell them apart if setting a consistent distance threshold.

In our case, we do not rely on coherence. We calculate the probability that a given dose causes recombination of more than one cell per embryo, by counting the frequency of bicolor specimens in the collection, by counting the number of cells recombined per embryo and by analyzing the live imaging data.

Minor comments
-please add the source of TAT-Cre : home-made or commercial ?
We have now added the reference for the TAT-cre in the Methods section:

“For fate mapping or local gene ablation experiments E7.5 to E8.5 embryos were microinjected with TAT-Cre recombinase **(SCR508, Sigma-Aldrich)**”

-Fig1E has red cells in the OFT : why is this not shown in Fig1B and not mentioned in the text ?

The embryo shown in Fig1E does not have red cells in the OFT. It may appear so because, when performing a maximal intensity projection of the stack of confocal optical planes, signal outside the heart tube located in other optical z-planes, overlaps with MF20 signal from the OFT.

This is an autofluorescent signal outside the OFT , which can be observed on the single optical planes (see figures below). The fact that it glows in both the green and red channels indicates it is autofluorescence, which sometimes occurs in whole-mount immunostained specimens.

© 2023. Published by The Company of Biologists under the terms of the Creative Commons Attribution License (https://creativecommons.org/licenses/by/4.0/).

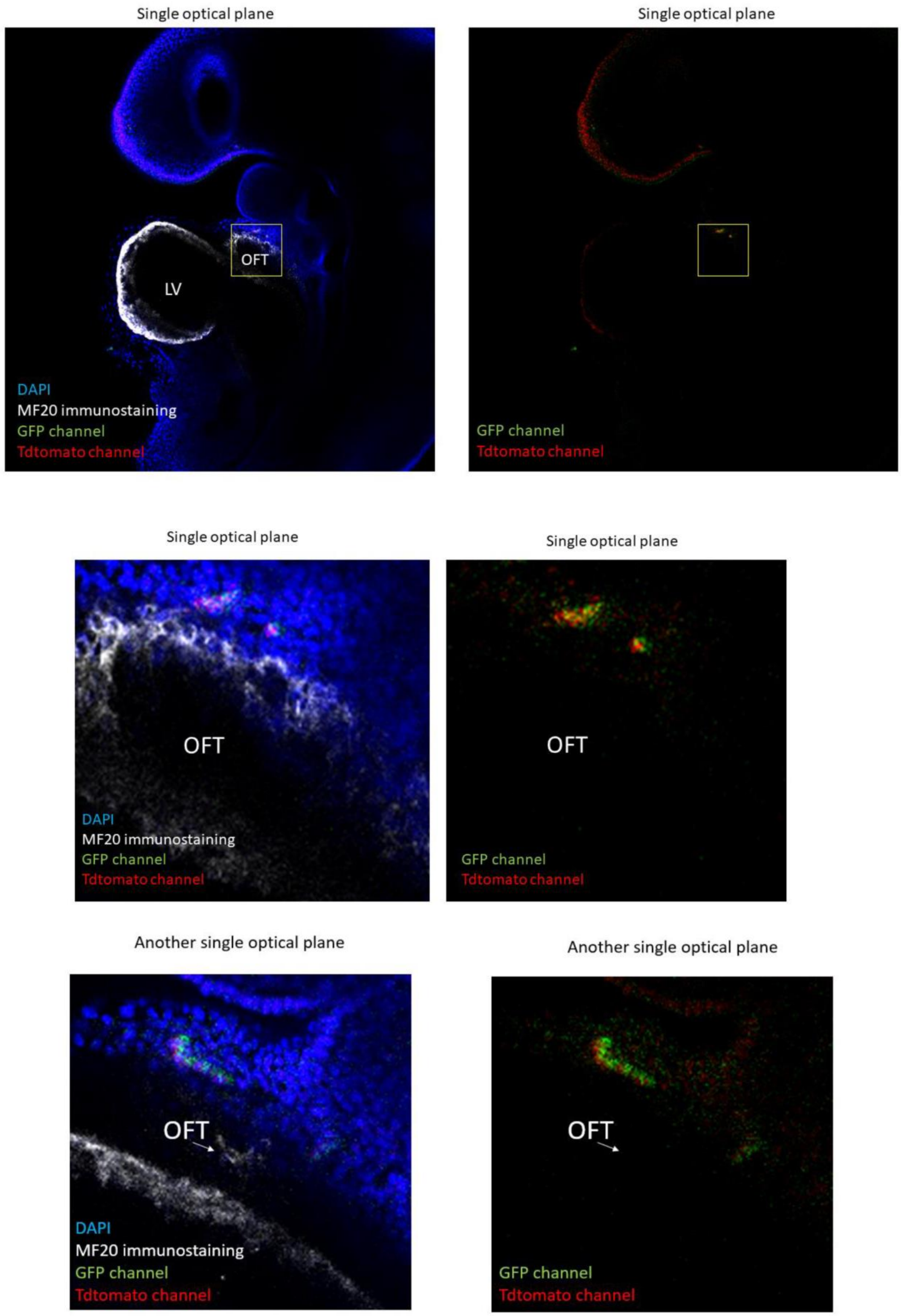

-In Fig3A : please specify the injection site of each clone and highlight clones considered in Fig 3D versus E

© 2023. Published by The Company of Biologists under the terms of the Creative Commons Attribution License (https://creativecommons.org/licenses/by/4.0/).

We added two extra columns to the right of Fig3A, each specifying the injection site and their consideration for Fig3D versus E. Clones are considered for Fig3D and E if they contain any of the cell types in each category. Thus, a clone containing both CM and PsM would be counted in both categories.

Cont
-annotate Fig.3 C, G, I to show tissues
We have now annotated Fig. 3 C, G and I following the abbreviations depicted in Fig3A.

-line 202-205 clarify your results compared to cited refs

We have now contextualized our results in more detail.

-line 210 : Tyser et al and Zhang et al don't show the primitive streak origin
We rephrased the sentence to make our point more clear:

"Interestingly, two of the clones contributed both to Embryonic and Extraembryonic compartments (Figure 3H and I); suggesting that the recently discovered multipotent progenitors (Tyser et al. 2021; Zhang et al. 2021) may be present in the early nascent mesoderm".

-typo "policlonal" and "fluorecent" Fig 2G-H, "early streak (MS)" Fig 3A, "pooled" and "pooler" FigS2
We thank the reviewer for pointing out these typos. We have corrected them in the new version.

Second decision letter

MS ID#: DEVELOP/2022/201206

MS TITLE: Cre recombinase microinjection for single-cell tracing and localised gene targeting

AUTHORS: Miquel Sendra, Juan de Dios Hourcade Bueno, Susana Temiño, Antonio José Sarabia, Oscar Horacio Ocaña, Jorge Nicolás Domínguez, and Miguel Torres

I have now received all the referees reports on the above manuscript, and have reached a decision. The referees' comments are appended below, or you can access them online: please go to BenchPress and click on the 'Manuscripts with Decisions' queue in the Author Area.

The overall evaluation is positive and we would like to publish a revised manuscript in Development, provided that the referees' comments can be satisfactorily addressed. Please attend to all of the reviewers' comments in your revised manuscript and detail them in your point-by-point response. If you do not agree with any of their criticisms or suggestions explain clearly why this is so. I am asking only for text revisions that I will evaluate without sending back to the reviewers.

Reviewer 2

Advance summary and potential significance to field

In this revised manuscript the authors have made an attempt to address my comments regarding their labelling approach. They have now included live-imaging and TAT-Cre (6xHis-Tag) immunostaining, which would address the concerns I previously raised however the sample size presented for the live-imaging is not sufficient and the manuscript requires a more thorough characterisation and reporting of the newly presented data. As previously stated, I believe this method could provide useful insight however describing exactly what is being labelled and whether it occurs in a reproducible manner is fundamental to make reliable conclusions.

© 2023. Published by The Company of Biologists under the terms of the Creative Commons Attribution License (https://creativecommons.org/licenses/by/4.0/).

Comments for the author

Figure S3A shows that only 2 embryos have been examined in which mesoderm was targeted with ½ dose, this is the main experimental protocol used therefore a larger sample size should be provided to support reproducibility and that only a single cell is labelled. Especially when statements are provided which suggest multiple cells could be labelled. “As for the question of whether cluster 8 and 9 in figure 3 correspond to multiple recombinations, we discuss that they may have originated from recombination in two cells from different germ layers” – how does this fit with the 2 live-imaging experiments which state that a single cell is labelled per embryo? This highlights the need for a significant increase in the number of embryos characterised.

How does the data presented in figure S3A fit with the graph in S3D. Which 5 embryos does this data refer to? If its ½ does then why are ectoderm cells labelled, does this reflect late recombination after live imaging? More thorough reporting is required to allow interpretation of the data presented.

Cell debris labelled in figure 2I only becomes fluorescent after 6:45h of culture, form the imaging provided how do the authors rule out that this isn’t a cell and another independent recombination event?

Figure S3A states that both mesoderm and ectoderm are aimed for with injection, although the proposed mechanism in S6B suggests that ectoderm is always targeted/labelled and mesoderm labelling is a by-product. This needs to be clarified given in the data presented, some embryos injected with the higher dose only label mesoderm how do these experiments fit with the proposed mechanism? Why is ectoderm being “aimed” for in these experiments, is this a control? How can the injector aim/assess whether the mesoderm or ectoderm is labelled given the brightfield imaging used for injection, the curved nature of mouse embryo and the force needed to puncture the endoderm.

To get a better insight into the feasibility of this approach it would help if the total number of embryos injected were provided. Do all embryos in which injections are attempted survive to analysis?

His-Tag characterisation has been conducted using the highest dose, I appreciate this represents the worst case scenario but why was ½ dose not the chosen condition given subsequent experiments and the previously provided insight. The images provided also don’t allow for any assessment in terms of which germ layer is being labelled. Do the cells shown in figure S4C reflect ectoderm or mesoderm expression?

What is the dose of Cre used in figure 1 and how does it compare to ½ and 1 dose?
“As the reviewer pointed out, clone 4 was injected in the proximal region (see Figure 3A)” – the figure in both 3A and B says distal, please clarify.
Figure 4S figure legends don’t match figure.

Reviewer 3

Advance summary and potential significance to field

The authors have well addressed my questions. They provide important new data by live-imaging and immunofluorescence of His-TAT-Cre and have clarified the text.

Overall this is an excellent paper providing a significant advance in the field of clonal analysis by establishing a new technique, which will be useful to a broad community of developmental biologits.

Comments for the author

I think the unspecific signal in Fig 1E should be annotated for all readers.

© 2023. Published by The Company of Biologists under the terms of the Creative Commons Attribution License (https://creativecommons.org/licenses/by/4.0/).

Given their explanations, I would suggest to avoid using the word cluster, which formally implies proximity (Oxford learner dictionary : "a group of things of the same type that grow or appear close together"), and is thus misleading. It could be replaced by "labelled cells".

Second revision

Author response to reviewers' comments

Reviewer 2 Advance Summary and Potential Significance to Field:

In this revised manuscript the authors have made an attempt to address my comments regarding their labelling approach. They have now included live-imaging and TAT–Cre (6xHis–Tag) immunostaining, which would address the concerns I previously raised however the sample size presented for the live-imaging is not sufficient and the manuscript requires a more thorough characterisation and reporting of the newly presented data. As previously stated, I believe this method could provide useful insight however describing exactly what is being labelled and whether it occurs in a reproducible manner is fundamental to make reliable conclusions.

We thank the reviewer for the comments. We explain below the reason for the limited N in the live imaging approach.

Reviewer 2 Comments for the Author:

Figure S3A shows that only 2 embryos have been examined in which mesoderm was targeted with ½ dose, this is the main experimental protocol used therefore a larger sample size should be provided to support reproducibility and that only a single cell is labelled. Especially when statements are provided which suggest multiple cells could be labelled. “As for the question of whether cluster 8 and 9 in figure 3 correspond to multiple recombinations, we discuss that they may have originated from recombination in two cells from different germ layers” – how does this fit with the 2 live-imaging experiments which state that a single cell is labelled per embryo? This highlights the need for a significant increase in the number of embryos characterised.

The demonstration that recombinations affect mostly single cells in these experiments does not derive from these two live-imaged specimens in which single cells were found to be labelled. The demonstration derives from the whole collection of fixed embryos analysed at the experimental endpoint, in which the frequency of embryos with two independent reporters was determined. As an additional proof of clonality, the number of labelled cells per embryo in correlation with the estimated cell division time was also used and in this revised version of the manuscript, this approach is validated by the direct observation of the cell cycle time. The live imaging was performed to demonstrate a) the timing of recombination, b) the tissue of recombination, c) the distribution of cells at the time of recombination with respect to the injection site, d) the division rate of the labelled cells. All these goals were fulfilled by live imaging of embryos injected with Cre concentrations that provoke polyclonal labelling, plus the 2 injected at concentrations that mostly generate clonal labels (total N of 7). The observations with the 2 embryos injected with the low dose have thus the qualitative value of showing that at this Cre dose it is possible to directly detect the clone-founder cell by live imaging. This is perfectly compatible with the possibility to find double recombination in some specimens when larger collections are examined (as clusters 8 and 9 in Figure 3), given the stochasticity of the recombination events. The important point is that the statistical analysis establishes the frequency of polyclonal recombination and the researcher can modulate the dose for reaching clonal resolution or choose to stay in the polyclonal range if clonality is not required in the experiments.

We would like to note the difficulty in obtaining these data in a microscope set up that allows to continuously image only one embryo at a time in experiments in which the researcher does not know which embryos will show recombined cells. This forces to either serially image several embryos one at a time or to monitor groups of embryos by manually acquiring time-lapse shots from each embryo at each timepoint to finally obtain data from a minority. The data presented

© 2023. Published by The Company of Biologists under the terms of the Creative Commons Attribution License (https://creativecommons.org/licenses/by/4.0/).

therefore already represent an experimental “tour de force” and we think extending these experiments would not add any essential information to the conclusions of the manuscript.

How does the data presented in figure S3A fit with the graph in S3D. Which 5 embryos does this data refer to? If its ½ does then why are ectoderm cells labelled, does this reflect late recombination after live imaging? More thorough reporting is required to allow interpretation of the data presented.

The data in graph S3D show cell counts obtained from tracking single cells to the culture endpoint (22 h) in live- imaged embryos shown in S3A. These include both Dose 1 and Dose ½ recombination events.

Only embryos e009 and e010 from S3A were not included in S3D, because identification of single cell progenies in these embryos was difficult (two or more recombination events with the same reporters). We have now indicated in S3A the embryos that were scored in S3D and explained why they were excluded.

Cell debris labelled in figure 2I only becomes fluorescent after 6:45h of culture, form the imaging provided how do the authors rule out that this isn’t a cell and another independent recombination event?

In this case, we cannot distinguish between an aborted recombination event and a dying cell that emits unspecific fluorescence. This type of signal appears as rounded particles that disaggregate and are pushed outside the embryo (never looking like healthy cells), which suggests the latter. Nonetheless, in experimental terms these events are not productive and therefore their nature does not modify the conclusions.

In the embryo shown in Figure 2I, the debris signal actually appears at 4:10 h, but at this early time it is outside the 20 μm stack that we projected in figure 2I. We now explain the timing of appearance of this signal in the Figure legend.

Figure S3A states that both mesoderm and ectoderm are aimed for with injection, although the proposed mechanism in S6B suggests that ectoderm is always targeted/labelled and mesoderm labelling is a by-product. This needs to be clarified given in the data presented, some embryos injected with the higher dose only label mesoderm how do these experiments fit with the proposed mechanism? Why is ectoderm being “aimed” for in these experiments, is this a control?

This should be a misunderstanding, as we did not state that mesoderm+ectoderm recombination was simultaneously aimed at any part of the manuscript.

On the contrary, we found that when the mesoderm is aimed (by injecting within this layer), the mesoderm is primarily recombined, but the ectoderm can occasionally recombine as a by-product (See figure 3, nascent mesoderm clonal analysis, 2 out of 19 injections produced off-target clones in the ectoderm). This is a limitation of the approach due to the diffusion of the TAT-Cre before recombination. We do not know if injections in the ectoderm can also cause occasional recombination in the mesoderm as a by-product. That would require additional clonal analyses focused in the ectoderm.

About S6B, this is an example of an injection aimed at the ectoderm to illustrate the proposed mechanism for recombination. It does not mean that injections aimed at the mesoderm also follow this mechanism. We have now modified the annotation of figure S6B to clarify that this is an “example for an ectoderm-aimed injection”.

How can the injector aim/assess whether the mesoderm or ectoderm is labelled given the brightfield imaging used for injection, the curved nature of mouse embryo and the force needed to puncture the endoderm.

Nomarsky contrast and micromanipulator control of the needle movements allows to visualize the embryonic layer targeted by the injections. The set up used is the same used for zygotic pronuclear microinjection. In the same way the pronuclei can be visualized and injected, we can determine the layer of injection in E6-E7 embryos.

© 2023. Published by The Company of Biologists under the terms of the Creative Commons Attribution License (https://creativecommons.org/licenses/by/4.0/).

To get a better insight into the feasibility of this approach it would help if the total number of embryos injected were provided. Do all embryos in which injections are attempted survive to analysis?

These data were provided in figure S7A, along with examples in S7B-D.

His-Tag characterisation has been conducted using the highest dose, I appreciate this represents the worst case scenario but why was ½ dose not the chosen condition given subsequent experiments and the previously provided insight.

As explained above, the number of recombined embryos at the low doses is scarce and makes experiments very difficult. We reasoned that doing the experiment at a higher dose would show the upper limits of the distribution range of the effectively injected cells. As the injected solution volume for dose ½ and dose 1 is the same, we reasoned that the higher dose of TAT-Cre would yield a clearer signal to noise ratio in the immunostaining while reporting an equivalent distribution of the injected solution. In other words, we are on the safe side by reporting distances of injected cells to the needle insertion site that in any case could be smaller if determined in lower- dose TAT-Cre injections, but never larger.

The images provided also don't allow for any assessment in terms of which germ layer is being labelled. Do the cells shown in figure S4C reflect ectoderm or mesoderm expression?

The single optical plane shown in S4C reveals signal in the ectoderm. We have now included this information in the figure legend.

What is the dose of Cre used in figure 1 and how does it compare to ½ and 1 dose?

We have now included this information in the Methods Section "TAT-Cre recombinase microinjection for fate mapping and gene ablation"

"As the reviewer pointed out, clone 4 was injected in the proximal region (see Figure 3A)" – the figure in both 3A and B says distal, please clarify.

This was a writing mistake when answering the reviewer. As the reviewer pointed out, clone 4 was injected in the *distal* region (see Figure 3A). The reviewer pointed out that both 3B and 3F injections appeared distal in terms of extension of the streak. In the first manuscript submitted, Figure 3B (clone 4) was correctly annotated in figure 3A but wrongly annotated in figure 3B. We corrected the annotation in 3B but mixed up the words when typing the answer to the reviewer.

Figure 4S figure legends don't match figure.

We revised the legend, and it is correct. We did not find any mismatch.

Reviewer 3 Advance Summary and Potential Significance to Field:
The authors have well addressed my questions. They provide important new data by live-imaging and immunofluorescence of His-TAT-Cre and have clarified the text.

Overall this is an excellent paper providing a significant advance in the field of clonal analysis by establishing a new technique, which will be useful to a broad community of developmental biologits.

We thank the reviewer for the appreciation of our work.

Reviewer 3 Comments for the Author:
I think the unspecific signal in Fig 1E should be annotated for all readers.

We have now annotated this in the Figure and its legend.

Given their explanations, I would suggest to avoid using the word cluster, which formally implies

© 2023. Published by The Company of Biologists under the terms of the Creative Commons Attribution License (https://creativecommons.org/licenses/by/4.0/).

proximity (Oxford learner dictionary : "a group of things of the same type that grow or appear close together"), and is thus misleading. It could be replaced by "labelled cells".

We agree with the reviewer and have corrected the text accordingly.

Third decision letter

MS ID#: DEVELOP/2022/201206

MS TITLE: Cre recombinase microinjection for single-cell tracing and localised gene targeting

AUTHORS: Miquel Sendra, Juan de Dios Hourcade Bueno, Susana Temiño, Antonio José Sarabia, Oscar Horacio Ocaña, Jorge Nicolás Domínguez, and Miguel Torres
ARTICLE TYPE: Techniques and Resources Report

I am happy to tell you that your manuscript has been accepted for publication in Development, pending our standard ethics checks.

© 2023. Published by The Company of Biologists under the terms of the Creative Commons Attribution License (https://creativecommons.org/licenses/by/4.0/).
